# A bispecific antibody approach for the potential prophylactic treatment of inherited bleeding disorders

**DOI:** 10.1038/s44161-023-00418-4

**Published:** 2024-02-08

**Authors:** Prafull S. Gandhi, Minka Zivkovic, Henrik Østergaard, Amalie C. Bonde, Torben Elm, Monika N. Løvgreen, Gerd Schluckebier, Eva Johansson, Ole H. Olsen, Eva H. N. Olsen, Ian-Arris de Bus, Karien Bloem, Oskar Alskär, Catherine J. Rea, Søren E. Bjørn, Roger E. Schutgens, Benny Sørensen, Rolf T. Urbanus, Johan H. Faber

**Affiliations:** 1 Hemab Therapeutics, Copenhagen, Denmark; 2 Center for Benign Haematology, Thrombosis and Haemostasis, Van Creveldkliniek, University Medical Center Utrecht, Utrecht University, Utrecht, Netherlands; 3 Novo Nordisk A/S, Måløv, Denmark; 4 Novo Nordisk Foundation Center for Basic Metabolic Research, University of Copenhagen, Copenhagen, Denmark; 5 EO Assay Consult, Ballerup, Denmark; 6 Sanquin Diagnostic Services, Amsterdam, Netherlands; 7 qPharmetra, Stockholm, Sweden; 8 Cymab Therapeutics, Copenhagen, Denmark

**Keywords:** Drug discovery, Pharmaceutics

## Abstract

Inherited bleeding disorders such as Glanzmann thrombasthenia (GT) lack prophylactic treatment options. As a result, serious bleeding episodes are treated acutely with blood product transfusions or frequent, repeated intravenous administration of recombinant activated coagulation factor VII (rFVIIa). Here we describe HMB-001, a bispecific antibody designed to bind and accumulate endogenous FVIIa and deliver it to sites of vascular injury by targeting it to the TREM (triggering receptor expressed on myeloid cells)-like transcript-1 (TLT-1) receptor that is selectively expressed on activated platelets. In healthy nonhuman primates, HMB-001 prolonged the half-life of endogenous FVIIa, resulting in its accumulation. Mouse bleeding studies confirmed antibody-mediated potentiation of FVIIa hemostatic activity by TLT-1 targeting. In ex vivo models of GT, HMB-001 localized FVIIa on activated platelets and potentiated fibrin-dependent platelet aggregation. Taken together, these results indicate that HMB-001 has the potential to offer subcutaneous prophylactic treatment to prevent bleeds in people with GT and other inherited bleeding disorders, with a low-frequency dosing regimen.

## Abstract (web summary)

Gandhi, Zivkovic, Østergaard and colleagues describe a bispecific antibody, HMB-001, which could be used for the prophylactic treatment of patients with genetic bleeding disorders, currently treated acutely with recombinant coagulation factor VIIa. HMB-001 can bind and accumulate endogenous FVIIa and localize it to sites of vascular injury by targeting it to the TREM-like transcript-1 receptor selectively expressed on activated platelets.

## Main

During the past seven decades, the treatment options for hemophilia A (HA) and B (HB) have advanced from transfusion of blood products to long-acting recombinant concentrates, to nonfactor therapies such as antibodies and small interfering RNAs, and now to gene therapy^1–3^. Consequently, the current standard of care for hemophilia treatment is largely focused on prophylaxis or preventing bleeding episodes. In contrast, many other serious bleeding disorders, including Glanzmann thrombasthenia (GT), coagulation factor VII (FVII) deficiency and Bernard–Soulier syndrome, completely lack prophylactic treatment options. As a result, patients experience frequent bleeds, which are treated acutely with approaches such as blood product transfusions or administration of prothrombin complex concentrates or recombinant activated FVII (rFVIIa)^4–6^.

**Fig. 1:**
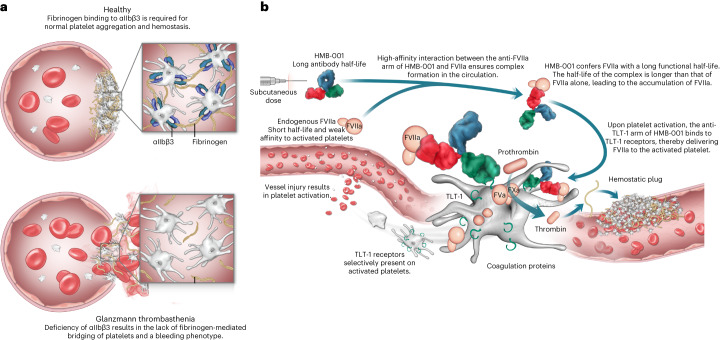
Proposed MoA of HMB-001. **a**, αIIbβ3 is a receptor for fibrinogen on platelets. In the case of normal platelets, fibrinogen binding to αIIbβ3 bridges platelets and is a required step for normal platelet aggregation and subsequent formation of a hemostatic plug. In the case of GT, deficiency of αIIbβ3 results in a lack of fibrinogen-mediated bridging of platelets, leading to abnormal platelet function manifesting in a severe bleeding phenotype. Due to the autosomal pattern of inheritance, GT affects both sexes equally. **b**, HMB-001 is a biAb designed to restore hemostasis through a mechanism mimicking that of rFVIIa but relying exclusively on the proteolytic activity of endogenous FVIIa. One arm of HMB-001 binds to endogenous FVIIa with high affinity. The half-life of HMB-001 is much longer than that of endogenous FVIIa. Therefore, the binding of HMB-001 to circulating FVIIa confers endogenous FVIIa with an extended half-life, resulting in progressive accumulation of plasma FVIIa until a new steady-state level is reached. The second arm of HMB-001 binds to the TLT-1 receptor on activated platelets. In the case of vascular injury, the binding of the anti-TLT-1 arm of HMB-001 to the TLT-1 receptor mediates increased recruitment of FVIIa onto the surface of the activated platelet. Here, HMB-001-delivered FVIIa drives FX activation and consequently enhances local thrombin generation to support the formation of a hemostatic plug. HMB-001 has the potential to offer subcutaneous-based prophylactic treatment, with a low frequency of dosing ranging from once a week to once a month, to prevent bleeds in PwGT and those with other rare bleeding disorders for which rFVIIa has been historically effective.

GT is an autosomal recessive disorder associated with a severe bleeding phenotype^7–9^. Approximately 50% of patients report experiencing one bleed daily and the negative impact of bleeding on their quality of life^10^. Mucocutaneous bleeding and heavy menstrual bleeding are the most commonly reported events^11,12^, and bleeds can be life-threatening. GT results from quantitative or qualitative mutations in the platelet membrane glycoprotein (GP) IIb/IIIa (αIIbβ3), which is essential for platelet aggregation and clot formation^9,13^ (Fig. 1a). Bleed management in people with GT (PwGT) is confounded by a lack of treatment options and evidence-based guidelines. Recommendations for managing minor bleeds include compression, elevation of the injured area, systemic tranexamic acid and topical application of antifibrinolytic agents^8,9,14,15^. For major bleeds and surgical interventions, one option is platelet transfusions. However, transfusions are often not readily available, especially for patients with anti-αIIbβ3 antibodies, and are complicated by the risk of alloimmunization, resulting in the person becoming refractory to further transfusions^8,9,14,15^. rFVIIa is licensed for acute bleed management in PwGT who are refractory to platelet transfusions and when platelets are unavailable^16^. It is also frequently used off-label to prevent surgical bleeds^9,17^. However, although effective in controlling bleeds, rFVIIa needs frequent intravenous infusions (several times per week) due to its short half-life^15,18^. The only other option is allogeneic human stem cell transplantation, which, although potentially curative, is associated with high mortality and morbidity risks^8,9,15,17^.

Mechanism of action (MoA) studies suggest that rFVIIa may function through a combination of tissue factor (TF)-independent and TF-dependent pathways^19–24^. In GT, it is proposed that rFVIIa drives factor X (FX) activation on the activated platelet surface in a TF-independent manner, generating an augmented thrombin burst at sites of vascular injury. rFVIIa-mediated thrombin generation results in fibrin formation on activated platelets, leading to fibrin-dependent but αIIbβ3-independent platelet aggregation and the formation of a hemostatic plug^13,20,25^.

rFVIIa has a very short systemic half-life (2–3 h), low subcutaneous bioavailability^26^ and weak binding affinity to activated platelets^27^. These features combined indicate the need for a dosing regimen of rFVIIa with multiple doses in the range of 90–270 μg kg^−1^ to treat an ongoing bleed, leading to peak plasma concentrations of 25–75 nM (ref. ^28^). Several rFVIIa analogues with varying degrees of backbone modifications were developed and investigated in preclinical and clinical studies to improve rFVIIa-based treatment^29–35^. These studies confirmed that the hemostatic properties of rFVIIa can be enhanced by improving its systemic half-life, activity or both. One strategy to improve FVIIa activity is by enhancing its affinity for activated platelets. This can be achieved by localizing FVIIa to a receptor on activated platelets^36^. Ideally, such a receptor should display high tissue specificity, be expressed selectively on activated platelets instead of resting platelets, be expressed abundantly on the surface and be amenable to therapeutic targeting without interfering with its intrinsic function.

TREM (triggering receptor expressed on myeloid cells)-like transcript-1 (TLT-1) fulfills each of these criteria. TLT-1 is a membrane-bound protein consisting of an extracellular N-terminal immunoglobulin variable (IgV) domain (amino acids 1–110) connected to a linker called the stalk peptide (amino acids 111–147), a transmembrane segment (amino acids 148–169) and a cytoplasmic domain (amino acids 170–296)^37^. TLT-1 is present exclusively in intracellular pools of resting platelets and megakaryocytes^38^. Upon platelet activation, TLT-1 is redistributed from α-granules to the platelet surface, exposing it to blood at the site of vascular injury^38,39^. Although TLT-1 has been demonstrated to bind fibrinogen, likely through its IgV domain, it appears to have a minor role in normal hemostasis, as TLT-1 knockout mice exhibit only a minor prolongation of bleeding time^40^.

A recent study demonstrated that FVIIa conjugated to an anti-TLT-1 Fab (fragment antigen-binding) fragment increased the affinity of FVIIa for activated platelets, leading to substantial enhancement of in vivo potency relative to free rFVIIa^41^. Building on the potential of targeted therapy and, at the same time, allowing for infrequent subcutaneous administration, we have designed a bispecific antibody (biAb) designated HMB-001. Using the DuoBody technology^42^ for biAb assembly, HMB-001 has been engineered to bind with high affinity to endogenous FVIIa with one arm and to TLT-1 on activated platelets with the second arm. Due to its long antibody half-life, HMB-001 confers endogenous FVIIa with a longer systemic half-life, resulting in the accumulation of HMB-001:FVIIa complexes in the circulation. Upon vascular injury, TLT-1 expression on activated platelets recruits HMB-001:FVIIa to the site of the lesion, resulting in enhanced FVIIa-driven hemostatic activity and potentiation of downstream thrombin generation and fibrin formation (Fig. 1b). Hence, HMB-001 builds upon the MoA of rFVIIa, except that it acts exclusively through endogenous FVIIa. In addition, HMB-001 serves as an example of antibody-mediated accumulation and targeting of an activated endogenous protein as a means of therapeutic intervention. Here, we describe the full characterization of HMB-001 and show that it results in improved hemostatic activity of FVIIa in ex vivo models of GT.

## Results

**Fig. 2:**
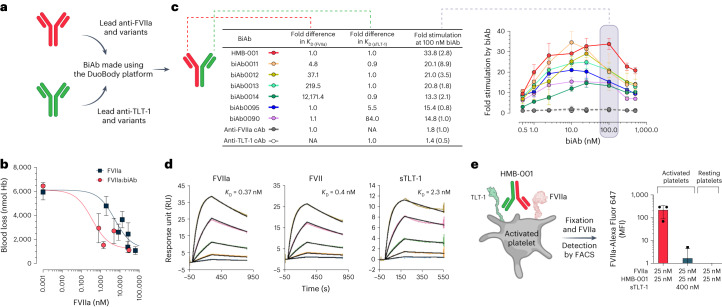
Optimal affinity for FVIIa and TLT-1 binding by HMB-001 leads to efficient TLT-1-dependent FVIIa localization on the activated platelet. **a**, BiAbs were made using the DuoBody platform. **b**, The ability of the biAb to potentiate FVIIa activity in vivo was evaluated in transgenic HA mice expressing human TLT-1 and using the TVT injury model. Anesthetized mice were placed on a heating pad set to maintain animal body temperature and with the tail submerged in saline (37 °C). FVIIa alone (*n* = 3–10, dark squares) or coformulated with an equimolar concentration of the biAb (*n* = 6, red circles) was administered intravenously into the right lateral tail vein 5 min before the injury. Total blood loss was determined by quantifying the amount of hemoglobin (Hb) in the saline and is expressed as nmol Hb. FVIIa concentrations were measured at the end of the bleeding window. Data are expressed as mean blood loss ± s.d. **c**, BiAbs with different affinities, as measured using surface plasmon resonance (SPR) technology (*n* = 2), toward FVIIa and sTLT-1 were generated. FX (150 nM) was activated with FVIIa (2.5 nM) in the presence of lipidated TLT-1 and biAbs at the indicated concentrations for 20 min (*n* = 3). Reactions were quenched, and FXa formation was assessed with a chromogenic substrate. At the anticipated clinically relevant biAb plasma concentration of 100 nM, shown in the shaded gray region, data are expressed as mean fold-stimulation in FXa generation compared to the absence of the biAb. Error bars indicate s.d. NA, not applicable. **d**, Binding of HMB-001 to FVIIa, zymogen FVII and sTLT-1 was assessed with SPR technology at 25 °C and pH 7.4 (*n* = 2). Kinetic data were fitted to a Langmuir 1:1 binding model to obtain *K*_D_ values. **e**, Whole blood from healthy donors (*n* = 3) was incubated with FVIIa, HMB-001 or sTLT-1, as indicated, in the presence of an Alexa Fluor 647-labeled FVIIa-specific V_H_H with or without a cocktail of 25 mM PAR-1 AP and 1 mg ml^−1^ CRP-XL for 10 min. FVIIa binding to platelets was assessed with FACS. Data are expressed as mean MFI ± s.d. Source data

**Extended Data Fig. 1:**
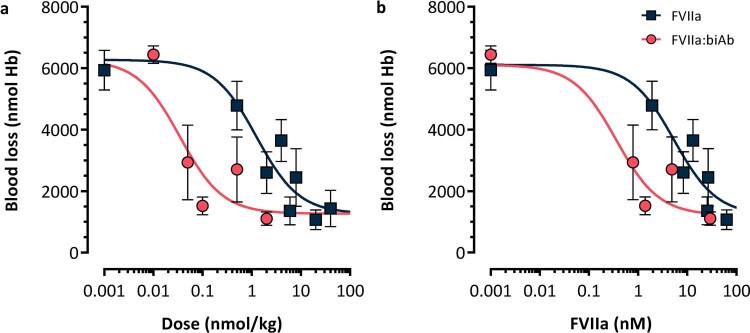
HMB-001 analogue biAb0097 reduces blood loss after tail vein transection in transgenic haemophilia A mice expressing human TLT-1. Mean blood loss as a function of **(a)** dose- and **(b)** plasma concentration of FVIIa following administration of FVIIa (n = 3–10, dark squares) or FVIIa coformulated with biAb0097 (1:1 mol/mol) (n = 6, red circles) in the tail vein transection model in HA mice carrying the human TLT-1 receptor. Data are mean blood loss ± s.d. FVIIa, activated factor VII; Hb, haemoglobin. Source data

**Extended Data Fig. 2:**
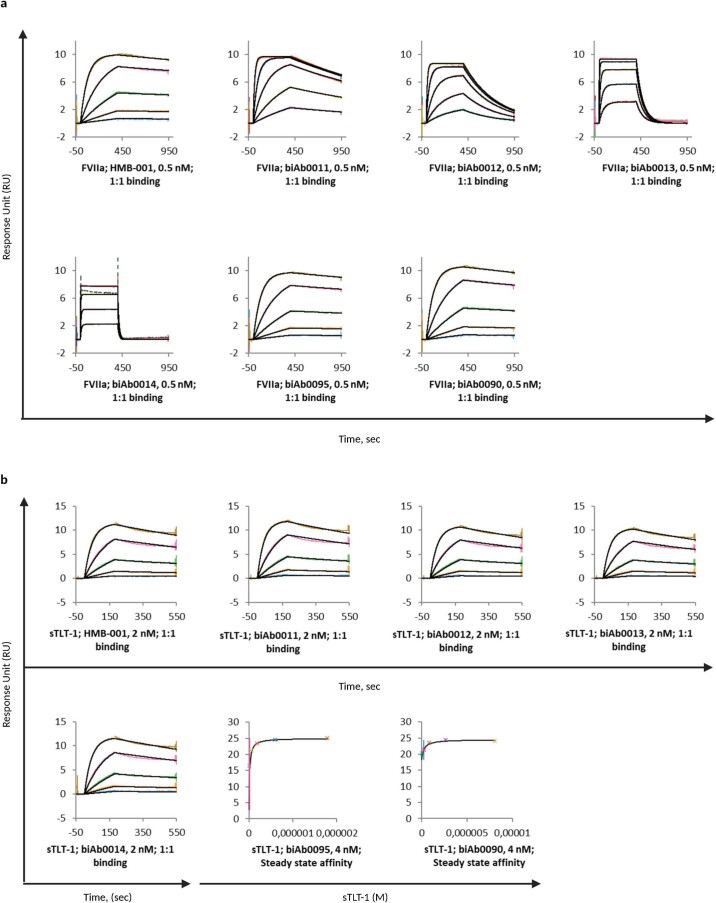
HMB-001 has the highest affinity for both FVIIa and sTLT-1. BiAbs (0.5–4 nM and as indicated on individual sensorgrams) were captured on immobilized anti-human IgG antibodies and binding of FVIIa (0–4 nM) (**a**) and sTLT-1 (0–60 mM) (**b**) was assessed with SPR at 25 °C and pH 7.4 (n = 2). Binding data were fitted to a Langmuir 1:1 binding model to determine association and dissociation rates as well as equilibrium dissociation constants (*K*_*D*_). Coloured lines represent binding data and solid black lines represent the fit of the data. Representative sensorgrams from one run are shown below. BiAbs, bispecific antibodies; IgG, immunoglobulin G, FVIIa, recombinant factor VIIa; SPR, surface plasmon resonance; sTLT-1, soluble extracellular fragment of TREM-like transcript 1. Source data

**Extended Data Fig. 3:**
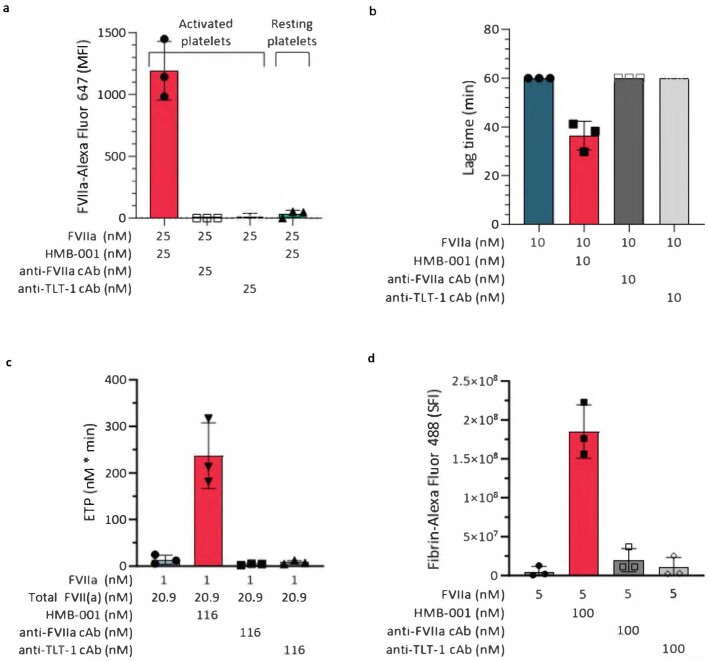
Effect of the two cAbs evaluated using different assays. **a**) Whole blood from healthy donors (n = 3) was incubated with FVIIa, HMB-001 or cAb as indicated in the presence of an AlexaFluor-647 labelled FVIIa-specific V_H_H with or without 25 mM PAR-1 AP and 1 mg/mL CRP-XL for 10 min at 37 °C. FVIIa binding to platelets was assessed with FACS. Data are expressed as mean MFI ± s.d. **b**) Fibrin-dependent platelet pseudoaggregation was measured in GT-like washed platelets in presence of 10 nM FVIIa, 0 or 10 nM HMB-001 and 0 or 10 nM cAb. Data are expressed as mean lag time ± s.d. (n = 3). **c**) Platelets were stimulated with both CRP-XL and PAR-1 AP, and thrombin generation was measured for 240 min in conditions mimicking scenario 3 (Table S8) (n = 3). FVIIa, Total FVII(a), HMB-001 or cAb was added as indicated. Bars represent mean endogenous thrombin potentials (ETP), error bars represent s.d. **d**) Area under the curve (AUC) of fibrin deposition in GT-like whole blood from healthy donors (n = 3) with 5 nM FVIIa and 0 or 100 nM HMB-001 or 0 or 100 nM cAb. Bars represent mean total fibrin formation in 20 min (sum of fluorescent intensity; SFI), error bars represent s.d. Source data

To identify anti-FVIIa and anti-TLT-1 antibodies for subsequent assembly into biAbs (Fig. 2a), we applied immunization and functional screening strategies to maintain the functionality of the two targets upon binding while allowing for productive assembly of the ternary FVIIa:biAb:TLT-1 complex on a procoagulant membrane surface. The anti-FVIIa arm of HMB-001 was selected based on the absence of effects on FVIIa-dependent thrombin generation and normal inhibition of FVIIa by antithrombin (AT). The anti-TLT-1 arm of HMB-001 was derived from the anti-TLT-1 Fab fragment already used for targeting FVIIa to activated platelets; this arm binds to the membrane-proximal stalk region of TLT-1 (ref. ^41^) and is anticipated not to affect the intrinsic properties of TLT-1. First, to determine whether targeting of FVIIa to TLT-1 on activated platelets with a biAb can potentiate FVIIa activity, we assembled an anti-FVIIa candidate and the lead anti-TLT-1 arm into a biAb and tested it in a tail vein transection (TVT) injury model in transgenic HA mice (*F8*^−/−^) expressing human TLT-1. FVIIa administration reduced blood loss after TVT in a concentration-dependent (Fig. 2b; half-maximal effective concentration (EC_50_) 5.4 nM, 95% confidence interval (95% CI) 1.7–14.3 nM) and dose-dependent manner (Extended Data Fig. 1). When FVIIa was coformulated with an equimolar amount of the biAb (to overcome the lack of cross-reactivity with murine FVIIa), the efficacy of FVIIa was enhanced by 15-fold (EC_50_ 0.36 nM, 95% CI 0.005–1.3 nM). The shorter half-life and, consequently, faster in vivo elimination of free FVIIa compared to the FVIIa:biAb complex were accounted for by estimating the EC_50_ values (Fig. 2b) based on the FVIIa concentrations measured in plasma at the end of the bleeding window. The improved efficacy of FVIIa in the presence of the biAb suggests that biAb-mediated targeting of FVIIa to platelet TLT-1 in vivo (Supplementary Table 1) is feasible. Next, several variants of the biAb with different target affinities were engineered to select optimal affinities for binding to FVIIa and TLT-1 (Fig. 2c, Extended Data Fig. 2 and Supplementary Table 2). The ability of each biAb to enhance TLT-1-dependent FVIIa activity was assessed in an FX activation assay (Fig. 2c). From this, the biAb with the highest affinity for FVIIa and the soluble extracellular fragment of TLT-1 (sTLT-1) was found to be associated with the greatest stimulation of FVIIa activity. Consequently, this candidate was selected as HMB-001. Subsequent titration of HMB-001 from 0 to 500 nM revealed a close-to-maximum potentiation of FVIIa activity in a broad concentration range encompassing HMB-001’s anticipated clinically relevant plasma concentration of 100 nM. In comparison, monovalent control antibodies (cAbs) comprising either the FVIIa- or TLT-1-binding arm of HMB-001 had no effect on FX activation, indicating that the combined action of both arms is required for the enhancing effect of HMB-001. HMB-001 bound to FVIIa, zymogen FVII and sTLT-1 with equilibrium dissociation constant (*K*_D_) values of 0.37, 0.4 and 2.3 nM, respectively (Fig. 2d and Supplementary Table 3). Consistent with the absence of TLT-1 on resting platelets, FVIIa localization was undetectable on resting platelets in the presence of HMB-001. In contrast, HMB-001 facilitated significant localization of FVIIa to platelets when these were preactivated with a cocktail of the protease-activated receptor-1 activating peptide (PAR-1 AP) SFLLRN and cross-linked collagen-related peptide (CRP-XL) (Fig. 2e). As expected, this was TLT-1 dependent, as demonstrated by a significantly impaired FVIIa localization when an excess (400 nM) of the competitor sTLT-1 was included in the assay. Neither the anti-FVIIa cAb nor the anti-TLT-1 cAb facilitated FVIIa binding (Extended Data Fig. 3a), demonstrating that FVIIa localization to activated platelets requires both arms of the HMB-001 biAb.

### HMB-001 does not affect the normal function of FVIIa or TLT-1

**Fig. 3:**
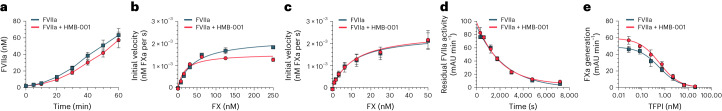
HMB-001 does not interfere with the normal functioning of FVIIa. **a**, FVII autoactivation. FVII (145 nM) was activated with FVIIa (2 nM) in the presence of lipidated TF (2 nM) and 0 or 500 nM HMB-001 for 0–60 min (*n* = 3). FVIIa activity was assessed with a chromogenic substrate in the presence of 200 nM lipidated TF. Data are expressed as mean ± s.d. **b**, TF-independent FX activation. Human plasma-derived FX (0–250 nM) was activated with FVIIa (20 nM) in the presence of 0 or 500 nM HMB-001 for 20 min (*n* = 3). FXa was assessed with a chromogenic substrate. FXa generation rates (nM FXa per s) were plotted as a function of FX concentration. Data are expressed as mean ± s.d. **c**, TF-dependent FX activation. Human plasma-derived FX (0–50 nM) was activated with FVIIa (100 pM) in the presence of 0 or 50 nM HMB-001 and 2 pM lipidated TF for 20 min (*n* = 3). FXa was assessed with a chromogenic substrate. FXa generation rates (nM FXa per s) were plotted as a function of FX concentration. Data are expressed as mean ± s.d. **d**, FVIIa inactivation by AT. FVIIa (200 nM) was preincubated with 12 mM low-molecular-weight heparin and 0 or 500 nM HMB-001 for 10 min, followed by incubation with AT for 0–2 h (*n* = 3). Residual FVIIa activity (mAU min^−1^) was assessed with a chromogenic substrate and plotted as a function of time. Data are expressed as mean ± s.d. **e**, FVIIa inactivation by TFPI. FVIIa (100 pM) was preincubated with 2 pM TF, 0–20 nM TFPI and 0 or 500 nM HMB-001 for 10 min, followed by incubation with 50 nM FX for 30 min (*n* = 3). FXa activity was assessed with a chromogenic substrate, and residual FXa activity (mAU min^−1^) was plotted as a function of TFPI concentration. Data are expressed as mean ± s.d. Source data

As maintaining the normal physiological functions of FVIIa by HMB-001 is crucial, we investigated the effects of HMB-001 on known reactions involving FVIIa or FVII. First, we addressed the impact of HMB-001 on the activation of downstream effectors of FVIIa^43,44^. HMB-001 did not affect FVII autoactivation or FX activation by TF-bound FVIIa but slightly enhanced FX activation by FVIIa in the absence of TF (Fig. 3a–c and Supplementary Table 4). Next, we analyzed the effect of HMB-001 on FVIIa inhibition. We observed little to no effect of HMB-001 on FVIIa inhibition by AT, which is responsible for elimination of rFVIIa from the circulation^45^, or tissue factor pathway inhibitor (TFPI), a key downregulator of TF-bound FVIIa during coagulation^46^ (Fig. 3d,e and Supplementary Table 4).

**Fig. 4:**
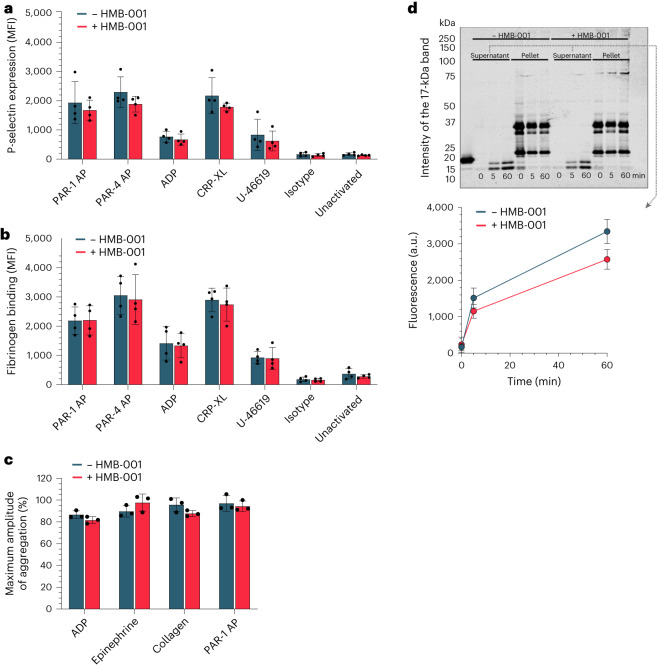
HMB-001 does not influence key platelet properties. **a**,**b**, Whole blood from healthy donors (*n* = 3) was incubated with 0 or 100 nM HMB-001 and buffer or platelet agonists (PAR-1 AP, PAR-4 AP, ADP, CRP-XL or U-46619) for 10 min. Platelet P-selectin expression (**a**) and fibrinogen binding (**b**) were measured with FACS. Data are expressed as mean MFI ± s.d. **c**, PRP from healthy donors (*n* = 3) was incubated with 0 or 100 nM HMB-001 and activated with ADP, epinephrine, collagen or PAR-1 AP with stirring at 900 r.p.m. Platelet aggregation was monitored with light transmission for 15 min. Data are expressed as the maximal amplitude of the aggregation trace and represent mean ± s.d. **d**, TLT-1 shedding. Washed platelets from healthy human donors (*n* = 3) were stimulated with collagen in an aggregometer for 0–60 min. Platelet fractions and supernatant were separated with centrifugation and subjected to SDS–PAGE. TLT-1 was visualized with western blotting. The intensity of the 17-kDa band was analyzed with Empiria Studio 2.1 software. Data represent mean ± s.d. Source data

**Extended Data Fig. 4:**
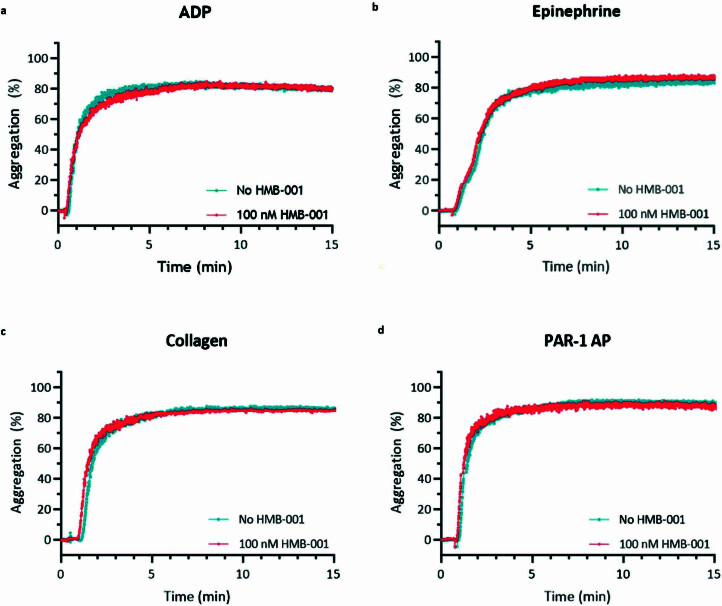
Effect of HMB-001 on normal platelet aggregation. Platelet rich plasma from healthy donors (n = 3) was incubated with 0 or 100 nM HMB-001 and activated with different activators while stirring at 900 rpm at 37 °C. Subsequent platelet aggregation was monitored with light transmission for 15 min. Shown are representative light transmission aggregometry traces in a single donor when activated with **a**) 5 µM ADP, **b**) 5 µM Epinephrine, **c**) 4 µg/mL Collagen, or **d**) 10 µM PAR-1 AP. Source data

**Extended Data Fig. 5:**
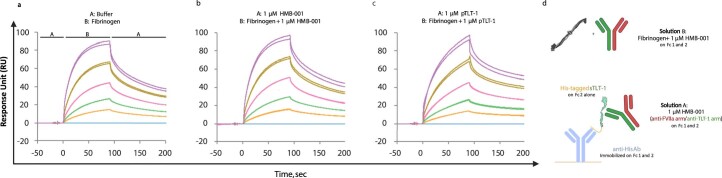
Fibrinogen binds to sTLT-1 in presence of HMB-001. Influence of **a**) Buffer, **b**) HMB-001, and **c**) parent TLT-1 Ab (pTLT-1) on the ability of fibrinogen to bind to sTLT-1 was probed using SPR at 25 °C and pH 7.4 (n = 2) using the ABA injection strategy. As shown in the SPR assay setup (**d**), sTLT-1 was captured using anti-His Ab. Solution A consisted of either running buffer or 1 μM HMB-001 or 1 μM pTLT-1 Ab. Solution B consisted of fibrinogen concentration series in presence of running buffer or 1 μM HMB-001 or 1 μM pTLT-1 Ab. Coloured lines represent different fibrinogen concentrations starting from the highest concentration of 1000 nM (purple), 500 nM (yellow), 250 nM (pink), 125 nM (green), 62.5 nM (orange), and 0 nM (blue). Sensorgram from both runs are overlaid and shown below. Source data

Furthermore, HMB-001 had no effect on platelet degranulation or activation, as evidenced by normal P-selectin secretion and fibrinogen binding after stimulation with agonists (Fig. 4a,b). In addition, HMB-001 did not influence the capacity of platelets to aggregate (Fig. 4c). Representative traces of platelet aggregation in a single donor with or without HMB-001 are shown in Extended Data Fig. 4. The biology of TLT-1 involves its partial downregulation through proteolytic cleavage and shedding on the activated platelet^47^. HMB-001 had no discernible effect on the shedding of TLT-1 (Fig. 4d) and allowed fibrinogen to engage with sTLT-1 (Extended Data Fig. 5). At saturating concentrations of HMB-001, its copy number per activated platelet ranged from 8,230 to 38,435, which is in line with previous reports of the number of TLT-1 copies per platelet^48,49^. Based on these data, HMB-001 was concluded to have little to no effect on normal FVIIa or platelet physiology.

**Fig. 5:**
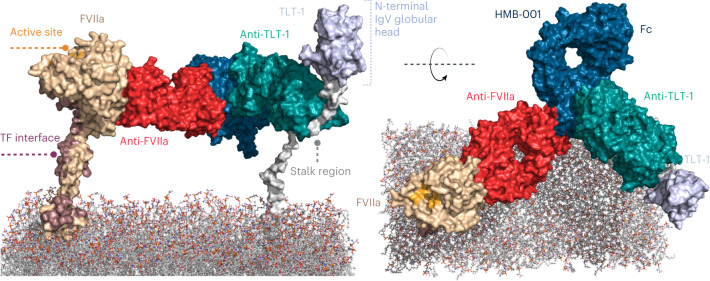
Ternary complex among HMB-001, FVIIa and TLT-1 on the phospholipid membrane surface. The model was generated by aligning (using PyMOL) the two crystal structures from the current study (PDB 8CN9 and 8CHE) with an antibody structure template (PDB 5DK3) and subsequently performing short molecular dynamics simulations on the initial starting structure.

**Extended Data Fig. 6:**
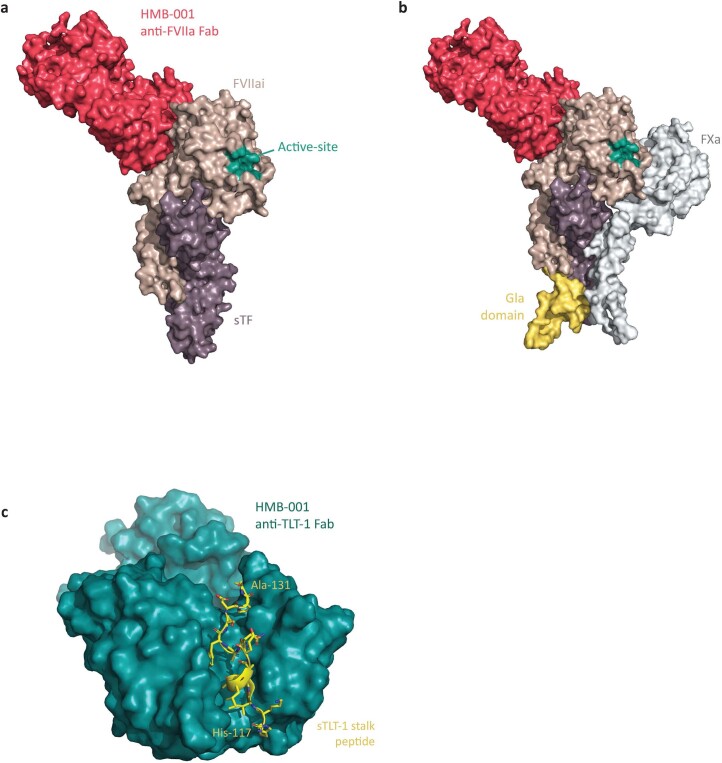
Structural overview. Crystal structures of Aa) anti-FVII Fab of HMB-001 bound to FVIIa:sTF complex, Bb) Addition of the predicted structure of the Gla domain of FVIIai (yellow colour) and FX/FXa (grey colour) based on an overlay of the crystal structure of FVIIai:sTF from:1DAN89 and a model of the FVIIa:sTF:FXa complex90, respectively and Cc) anti-TLT-1 Fab of HMB-001 bound to sTLT-1 stalk peptide. FVIIai, active-site inhibited FVIIa-desGLA; sTF, soluble fragment of tissue factor; Gla domain, N-terminal γ-carboxyglutamic domain; FXa, coagulation activated factor X; sTLT-1, soluble fragment of TREM-like transcript 1.

To characterize the binding epitopes of HMB-001 on FVIIa and TLT-1, we solved the x-ray crystal structures of the anti-FVIIa Fab fragment in complex with the active site-inhibited FVIIa:soluble TF (sTF) complex (Protein Data Bank (PDB) 8CN9) and the anti-TLT-1 Fab fragment in complex with the synthetic stalk peptide of TLT-1 (PDB 8CHE). The data collection and refinement statistics are summarized in Supplementary Table 5. The crystal structure of the anti-FVIIa Fab in complex with the active site-inhibited FVIIa:sTF complex (Extended Data Fig. 6a) showed that the anti-FVIIa arm of HMB-001 recognizes an epitope on FVIIa composed of residues from the epidermal growth factor-like 2 and protease domains of FVIIa. The binding epitope does not overlap with the active site of FVIIa, the binding interface with TF or the N-terminal γ-carboxyglutamic acid (Gla) domain of FVIIa that is involved in membrane binding. A comparison to the published molecular model of the FVIIa:sTF:activated FX (FXa) complex further suggests that the epitope is distinct from the predicted binding site of FX/FXa (Extended Data Fig. 6b), consistent with functional studies. The crystal structure of the anti-TLT-1 Fab in complex with the TLT-1 stalk peptide (Extended Data Fig. 6c) showed that this arm of HMB-001 recognizes a linear segment in the stalk region comprising amino acids from K118 to A131 (mature TLT-1 receptor numbering). Consequently, the binding epitope is distant from the N-terminal globular IgV domain and the transmembrane sequence of the TLT-1 receptor. By using the two crystal structures, a model of the ternary complex among HMB-001, FVIIa and TLT-1 receptor embedded in the phospholipid membrane was generated (Fig. 5). Consistent with the two crystal structures and available functional data, the generated model supports the lack of interference of HMB-001 with the known functional properties of FVIIa and TLT-1. In addition, the orientation of the HMB-001:FVIIa complex with respect to the HMB-001:TLT-1 receptor complex and the fragment crystallizable (Fc) domain of HMB-001 allows HMB-001 to simultaneously engage with FVIIa and TLT-1, while the FVIIa N-terminal Gla domain is free to dock in an apparent productive orientation on the phospholipid membrane surface^50^.

### HMB-001 accumulates endogenous FVIIa in nonhuman primates

**Fig. 6:**
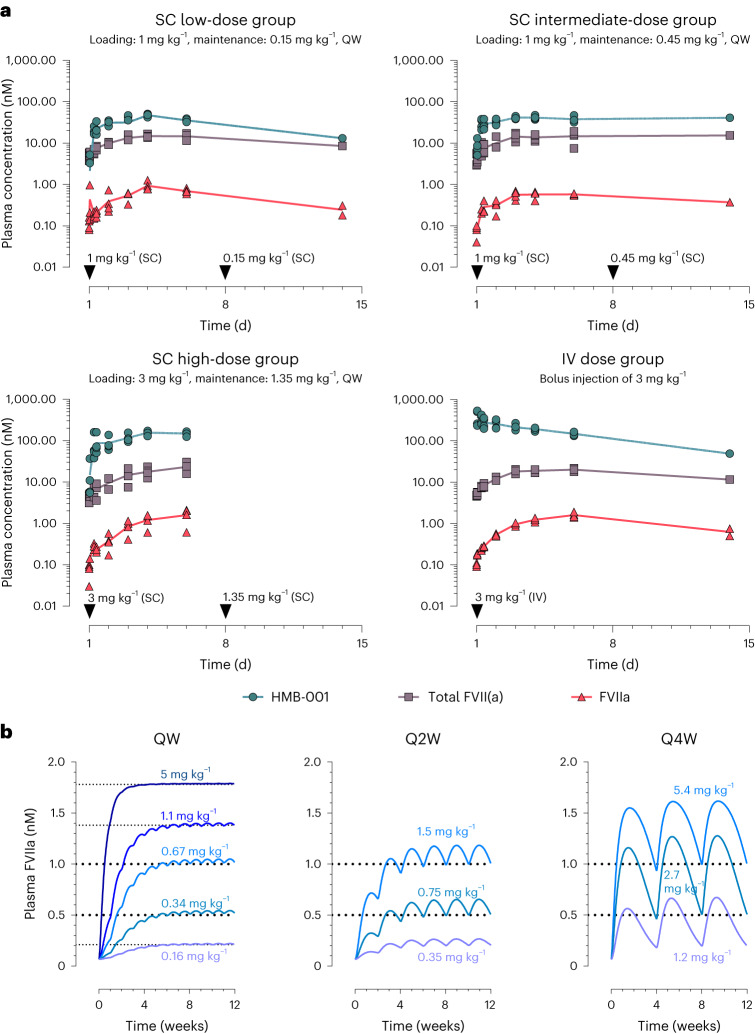
HMB-001 leads to time- and dose-dependent accumulation of endogenous FVIIa and total FVII(a). **a**, PK results of HMB-001 and time course of FVIIa and total FVII(a) accumulation in cynomolgus monkeys following subcutaneous (SC) and intravenous (IV) administration of HMB-001. Three groups (*n* = 4) were administered HMB-001 subcutaneously once weekly (QW), with clinically relevant loading and maintenance doses of 1 and 0.15, 1 and 0.45, and 3 and 1.35 mg kg^−1^, respectively. A fourth group (*n* = 4) was administered an intravenous bolus injection of 3 mg kg^−1^ HMB-001. Measured plasma concentrations of FVIIa (red triangles), total FVII(a) (gray squares) and HMB-001 (green circles) are shown for individual animals. For clarity purposes, only data points from ADA-negative plasma samples are shown. Solid lines connect the calculated means for each time point. Cynomolgus monkey FVIIa and total FVII(a) were measured using modified human FVIIa clot activity and human FVII ELISA kits (Stago) and, hence, are referred to as human-equivalent levels (Methods). **b**, Simulation of multiple-dose subcutaneous administration of HMB-001 in humans using a PK/PD model scaled to the human setting. For once-weekly simulations, five different clinical scenarios were simulated to identify the HMB-001 once-weekly dose to reach target accumulated endogenous FVIIa levels of 0.21, 0.52, 1, 1.38 and 1.78 nM (shown by horizontal dotted lines). Corresponding levels of total FVII(a) and HMB-001 are summarized in Supplementary Table 8. Every-2-week (Q2W) and every-4-week (Q4W) simulations were undertaken to show that endogenous FVIIa can be accumulated to target levels of 0.5 and 1 nM (horizontal dotted lines) with less frequent dosing regimens. HMB-001 doses predicted to be needed to reach each target FVIIa level are shown in blue and purple. Source data

To assess the pharmacokinetic (PK) properties of HMB-001 and probe its ability to accumulate endogenous FVIIa, a measure of HMB-001 pharmacodynamic (PD) activity, we carried out a study in cynomolgus monkeys. In all animals, we observed a dose- and time-dependent accumulation of endogenous FVIIa and total FVII(a) (that is, total FVII including zymogen FVII, FVIIa and other forms of FVIIa (for example, inhibited forms of FVIIa)) (Fig. 6a). The predose and maximum plasma concentrations (*C*_max_) of FVIIa and total FVII(a) for each group are summarized in Supplementary Table 6. In the group receiving the highest subcutaneous dose of HMB-001, FVIIa increased up to 20-fold and total FVII(a) increased up to 5.6-fold relative to the predose levels. At *C*_max_ on day 6 for this group, FVIIa and total FVII(a) reached mean levels of 1.6 and 23.3 nM, respectively, compared to the predose levels of 0.08 and 4.2 nM, respectively. All animals completed the study. In addition, no significant changes in platelet counts were observed (Supplementary Table 7a), and the change in body weight was as expected (Supplementary Table 7b). Based on the obtained PK results, a PK/PD model was built and scaled to the human situation using allometric principles (Methods). As shown in Fig. 6b, the PK/PD model predicts that endogenous FVIIa concentrations in the low-nM range can be reached in a dose-dependent fashion following subcutaneous administration of HMB-001 in humans, with a dosing frequency of once weekly to once monthly.

### Potential of HMB-001 to treat GT

**Fig. 7:**
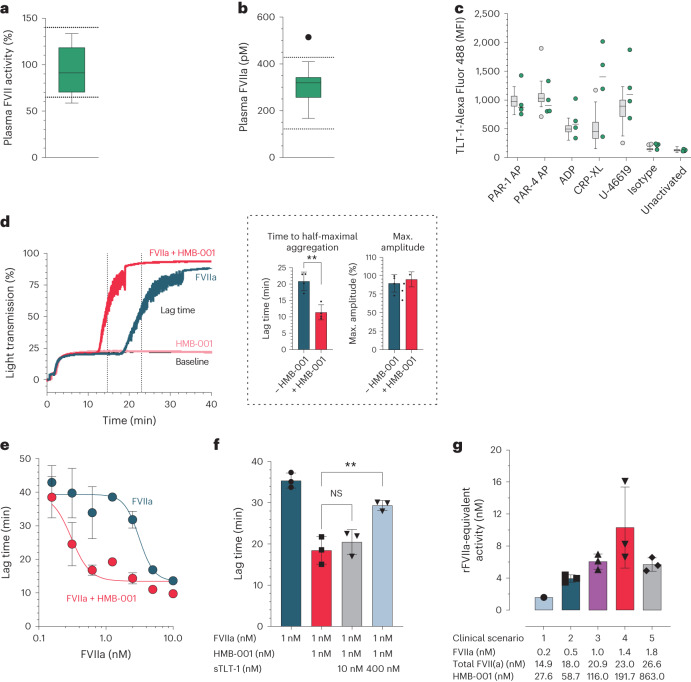
HMB-001 enhances FVIIa-dependent thrombin generation and aggregation in GT platelets. **a**, Plasma FVII activity was assessed with a PT-based activity assay in 13 PwGT. Box plot represents the interquartile range. Horizontal line represents the median. Whiskers represent the upper and lower adjacent values. Dotted lines represent the local hospital reference range. **b**, Plasma FVIIa levels were measured with ELISA in 13 PwGT. Box plot represents the interquartile range. Horizontal line represents the median. Whiskers represent the upper and lower adjacent values. Outliers are indicated as filled black circles. Dotted lines represent the 2.5th and 97.5th percentiles of the FVIIa levels in 50 healthy controls. **c**, TLT-1 expression was measured with FACS in whole blood of 4 PwGT (green) and 51 healthy controls (gray) in resting platelets or after stimulation with agonists. A labeled IgG4 was used as an isotype control. Box plots represent the interquartile range. Horizontal lines represent the median. Whiskers represent the upper and lower adjacent values. Outliers are shown as filled grey circles. **d**, Fibrin-dependent platelet pseudoaggregation was measured in four PwGT. Light transmission was monitored for 40 min. Shown are representative traces of light transmission aggregometry in a PwGT (left) and the mean ± s.d. of lag time and the maximum amplitude of the aggregation traces with and without HMB-001 (right, inside the dashed box). Lag time is defined as the time to half-maximal aggregation and is indicated by the vertical dotted lines. Data were analyzed with a two-sided unpaired *t* test (**P = 0.0019). **e**, Fibrin-dependent platelet pseudoaggregation was measured in washed platelets from healthy controls supplemented with d-RGDW to obtain GT-like platelets (*n* = 3 for each concentration). Aggregation lag time was determined in the presence of FVIIa with or without HMB-001. Data represent mean ± s.d. aggregation lag time at the indicated FVIIa concentration. **f**, Fibrin-dependent platelet pseudoaggregation was measured in GT-like washed platelets in the presence of 1 nM FVIIa; 0 or 1 nM HMB-001; and 0, 10 or 400 nM sTLT-1. Data are expressed as mean lag time ± s.d. (*n* = 3). Data were analyzed with a two-sided unpaired *t* test (**P = 0.0061). NS, not significant. **g**, rFVIIa-equivalent activity was measured in PRP from healthy controls (*n* = 3) supplemented with d-RGDW to obtain GT-like platelets and a blocking anti-TF antibody to ensure TF independence. FVIIa, FVII and HMB-001 were added at the concentrations obtained by simulating five clinical scenarios (Supplementary Table 8). Bars represent the mean rFVIIa-equivalent activity; error bars represent s.d. Source data

The presence of circulating FVIIa and the expression of TLT-1 upon platelet activation are essential for the MoA of HMB-001. To explore the potential of HMB-001 to treat GT, we quantified FVII activity, FVIIa levels and platelet TLT-1 expression in PwGT and healthy volunteers. FVII activity and FVIIa plasma levels were similar in healthy volunteers and PwGT (Fig. 7a,b). Similar to P-selectin, TLT-1 was not expressed on resting platelets but was induced to a similar degree on healthy and GT platelets after stimulation with a panel of agonists (Fig. 7c).

Fibrinogen-dependent platelet aggregation is absent in GT, but platelet aggregation responses can occur when fibrin formation is allowed, for example, by rFVIIa^13^. This aggregation response reflects platelet capture in a fibrin mesh^15^, which we will refer to as fibrin-dependent pseudoaggregation. To determine whether HMB-001 potentiates FVIIa activity on activated GT platelets, we stimulated washed GT platelets with collagen in the presence of FVIIa, FX, prothrombin and fibrinogen to allow fibrin formation. In the absence of FVIIa or the presence of HMB-001 alone, no fibrin-dependent pseudoaggregation occurred after collagen stimulation, demonstrating the complete absence of the fibrinogen receptor. Addition of 10 nM FVIIa resulted in complete fibrin-dependent pseudoaggregation after a mean (s.d.) lag time of 20.9 (2.8) min (Fig. 7d), consistent with the ability of rFVIIa to induce fibrin-dependent platelet pseudoaggregation in GT^13^. Fibrin-dependent pseudoaggregation was potentiated when FVIIa was supplemented with equimolar concentrations of HMB-001 (10 nM each), resulting in a mean (s.d.) lag time of 11.4 (2.3) min.

Next, the relationship between FVIIa concentration and lag time was investigated in the presence and absence of an equimolar concentration of HMB-001 under GT-like conditions. To mimic GT conditions, we incubated washed platelets from healthy donors with the d-RGDW^25^ antagonist peptide to inhibit the fibrinogen receptor. While the mean (s.d.) EC_50_ value for FVIIa alone was 3.12 (0.75) nM, the mean (s.d.) EC_50_ value decreased to 0.31 (12) nM in the presence of HMB-001, representing a tenfold potentiation by HMB-001 (Fig. 7e). Monovalent cAbs did not potentiate fibrin-dependent pseudoaggregation (Extended Data Fig. 3b). To verify the TLT-1 dependency of the potentiating activity of HMB-001, we performed aggregation experiments in the presence of the competitor sTLT-1, which indeed antagonized the effect of HMB-001, confirming the TLT-1-dependent MoA of HMB-001 (Fig. 7f).

**Extended Data Fig. 7:**
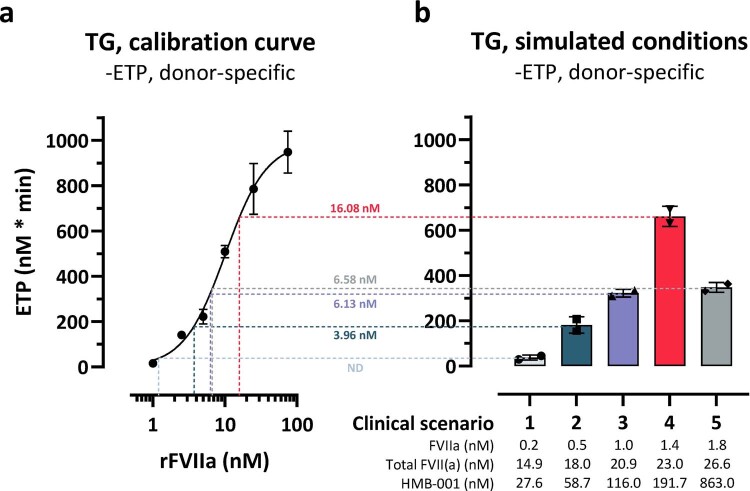
Representative data from a single donor showing enhanced FVIIa activity by HMB-001 at predicted plasma levels of FVIIa, total FVII(a) and HMB-001 following five different dosing regimens. rFVIIa-equivalent activity was measured in PRP from a healthy control (n = 3) supplemented with d-RGDW to obtain GT-like platelets and a blocking anti-TF antibody to ensure TF-independence. PRP was supplemented with rFVIIa (0–75 nM), platelets were stimulated with CRP-XL and PAR-1 AP, and thrombin generation was measured for 240 min. **a)** For each donor, Endogenous thrombin potential (ETP) was determined in duplicate as a function of rFVIIa concentration. Data represent mean ± s.d. **b)** ETP was determined in duplicate at 5 predicted clinical scenarios of FVIIa, total FVII(a) and HMB-001 in healthy controls (n = 3). The background plasma concentrations of FVIIa and total FVII(a) were assumed to be 67 pM and 10 nM, respectively. Predicted accumulated levels of total FVII(a) and FVIIa for 5 predicted clinical scenarios (Table S8) were reconstituted by adding FVII-S195A and FVIIa after accounting for the background plasma concentration of total FVII(a) and FVIIa respectively. HMB-001 was added as indicated. Data represent mean ± s.d. For each clinical scenario, the corresponding rFVIIa-equivalent activity (nM), as extrapolated from the X-axis of the calibration curve, is indicated next to the horizontal dotted line for the single donor. rFVIIa-equivalent activities for three donors are shown in Fig. 7G. Source data

**Extended Data Fig. 8:**
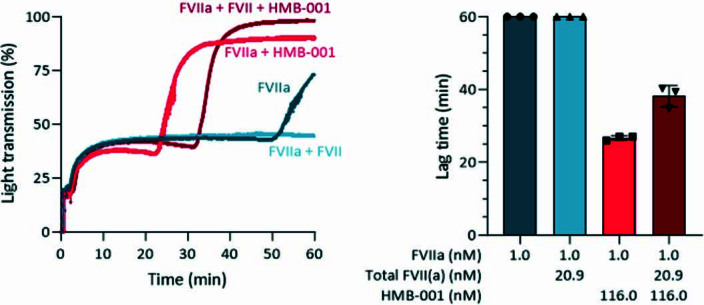
HMB-001 enhances fibrin dependent platelet aggregation at predicted plasma levels of FVIIa, total FVII(a) and HMB-001 following a once weekly HMB-001 dose of 0.67 mg/kg. Fibrin-dependent platelet pseudoaggregation was measured in washed platelets from healthy donors supplemented with d-RGDW (GT-like; n = 3). The data in the bar chart are expressed as mean ± s.d. Aggregation was induced with collagen in buffer with 3 mM CaCl2+, 10 mg/mL FX, 20 ng/mL prothrombin and 0.5 mg/mL fibrinogen. The background plasma concentrations of FVIIa and total FVII(a) were assumed to be 67 pM and 10 nM, respectively. Predicted accumulated levels of total FVII(a) and FVIIa for once weekly HMB-001 dose of 0.67 mg/kg (scenario 3, Table S8) were reconstituted by adding FVII-S195A and FVIIa after accounting for the background plasma concentration of total FVII(a) and FVIIa respectively. HMB-001 was added as indicated. Light transmission was monitored for 60 min. Shown are representative light transmission aggregometry traces in a single donor, as well as summary data on fibrin-dependent pseudoaggregation lag time in all donors. Source data

Next, we determined whether HMB-001 has the potential to bring the activity of accumulated endogenous FVIIa and total FVII(a) to levels approaching the range of rFVIIa that is considered efficacious in treating acute bleeds in PwGT. PK/PD studies in cynomolgus monkeys indicated that HMB-001 accumulates both FVIIa and total FVII(a). To compare the efficacy of HMB-001-mediated accumulation of FVIIa and total FVII(a) to that of rFVIIa alone, we analyzed thrombin generation in platelet-rich plasma (PRP) under HA- and GT-like conditions using five different simulated clinical scenarios of HMB-001 administration (Fig. 6b and Supplementary Table 8). By comparing the endogenous thrombin potential observed in each modeled clinical scenario to the response obtained with rFVIIa alone, an rFVIIa-equivalent activity for each scenario was calculated (Fig. 7g and Extended Data Fig. 7). The rFVIIa-equivalent activity ranged from 1.6 to 10.3 nM and was dose dependent, except at the highest concentration of HMB-001 tested (863 nM, scenario 5), in which a decline in rFVIIa-equivalent activity from 10.3 to 5.7 nM was observed. This is likely due to the competition between the excess free HMB-001 and HMB-001:FVIIa for binding to TLT-1 receptors on the activated platelets. Monovalent cAbs did not enhance FVIIa activity (Extended Data Fig. 3c). At FVIIa and total FVII(a) levels predicted to be present during HMB-001 treatment, HMB-001 also potentiated the fibrin-dependent pseudoaggregation of GT-like platelets (Extended Data Fig. 8).

**Fig. 8:**
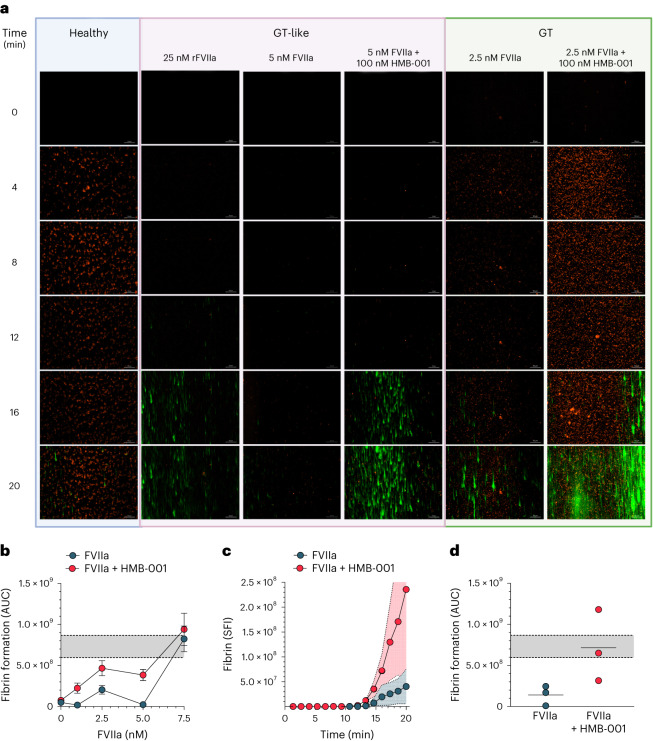
HMB-001 enhances fibrin formation on adhered GT platelets in flowing blood. **a**–**d**, Recalcified whole blood was perfused over a collagen-coated surface in a microfluidic device at a shear rate of 300 s^−1^. Platelets were labeled with MitoTracker Orange CMTMRos, and fibrin was detected with an Alexa Fluor 488-conjugated V_H_H antifibrin antibody. Platelet adhesion and fibrin deposition were monitored in whole blood from healthy controls, whole blood from healthy controls supplemented with d-RGDW (GT-like), and whole blood from PwGT. Platelet adhesion and fibrin deposition were monitored for 20 min at a frame rate of three per minute using a Zeiss Observer Z1 widefield fluorescence microscope with Colibri LEDs at 200-fold magnification. **a**, Representative time-lapse of platelet adhesion (orange) and fibrin deposition (green) in blood from healthy controls; GT-like whole blood with 25 nM rFVIIa, 5 nM FVIIa, and 0 or 100 nM HMB-001; and whole blood from a PwGT supplemented with 2.5 nM FVIIa and 0 or 100 nM HMB-001. **b**, Fibrin deposition was quantified using ZEN 2 (blue edition) software. Data are expressed as the area under the curve (AUC) of fibrin deposition on platelets adhered to collagen in GT-like whole blood supplemented with 0–7.5 nM FVIIa and 0 or 100 nM HMB-001 (*n* = 3–7 for each concentration). Gray area between dotted lines represents the mean area under the curve ± s.d. of fibrin deposition in GT-like whole blood supplemented with 25 nM rFVIIa (*n* = 7). **c**, Fibrin deposition on platelets adhered to collagen after the perfusion of whole blood from PwGT (*n* = 3) supplemented with 2.5 nM FVIIa and 0 or 100 nM HMB-001. Data are expressed as the mean sum of fluorescence intensity on each frame (SFI) as a function of time. Shaded areas indicate s.d. **d**, Area under the curve of fibrin deposition in whole blood from PwGT (*n* = 3) supplemented with 0 or 100 nM HMB-001. Gray area between dotted lines represents the mean area under the curve ± s.d. of fibrin deposition in GT-like whole blood supplemented with 25 nM rFVIIa (*n* = 7). Source data

Having determined the ability of HMB-001 to potentiate FVIIa activity under static conditions, we next evaluated the efficacy of HMB-001 in a model of GT through a microfluidic assay, which allows for the evaluation of platelet adhesion and fibrin formation in flowing blood and has been used extensively to evaluate thrombus formation in bleeding disorders^51^. For example, in one such assay, FVIIa enhanced thrombin generation on GT platelets adhered to collagen in the absence of TF^25^. We perfused whole blood over a collagen-coated surface and monitored platelet adhesion and fibrin deposition. In blood from healthy controls, platelet aggregation on the collagen surface occurred rapidly with little fibrin deposition observed after 20 min (Fig. 8a). Under GT-like conditions, supplementation of FVIIa resulted in a dose-dependent increase in fibrin deposition (Fig. 8b). Substantial fibrin deposition on adhered GT-like platelets was seen with 25 nM rFVIIa (Fig. 8a), which corresponds to the peak FVIIa plasma concentration reached after a therapeutic dose of 90 μg kg^−1^ rFVIIa^28^. The addition of 100 nM HMB-001 potentiated the effect of FVIIa at all concentrations tested in the range from 0 to 7.5 nM FVIIa (Fig. 8b). At 5 nM FVIIa, fibrin deposition in the presence of 100 nM HMB-001 was higher than that achieved with 25 nM rFVIIa alone. In contrast, fibrin deposition in the presence of 100 nM monovalent cAbs was similar to fibrin deposition with 5 nM FVIIa alone (Extended Data Fig. 3d). These results were confirmed in whole blood from three PwGT. Here, fibrin deposition at 2.5 nM FVIIa and 100 nM HMB-001 was similar to that achieved with 25 nM rFVIIa alone in GT-like conditions (Fig. 8a,c,d).

## Discussion

HMB-001 is a biAb that binds FVIIa and TLT-1 and is designed to restore hemostasis through a mechanism mimicking that of rFVIIa but relying exclusively on the proteolytic activity of endogenous FVIIa. Here, we have shown that HMB-001 does not interfere with the normal functioning of FVIIa or influence key platelet properties while effectively targeting FVIIa to the activated platelet surface. Subcutaneous dosing of HMB-001 in cynomolgus monkeys resulted in a dose- and time-dependent accumulation of endogenous FVIIa to the low-nM range (0.6–1.6 nM). Experiments in ex vivo GT models showed that HMB-001 enhanced the effect of FVIIa on fibrin-dependent platelet pseudoaggregation by approximately tenfold (Fig. 7e) and substantially improved fibrin formation on adhered GT platelets in flowing blood. Using an analogue of HMB-001, in vivo studies in a mouse bleeding model showed a similar 15-fold potency enhancement of FVIIa activity (Fig. 2b).

Structural studies revealed that HMB-001 engages FVIIa at an epitope distant from known physiologically important surface patches, providing a rationale for the observed lack of effect in non-cell-based functional studies. In addition, the minor role of TLT-1 in hemostasis^40^ combined with the location of the binding epitope in the stalk region of TLT-1 ensures that HMB-001 does not influence platelet properties and TLT-1 function. The lack of effect of HMB-001 on platelet physiology supports this notion. Taken together, these results support the intended mechanism involving high-affinity binding and dose-dependent accumulation of endogenous FVIIa and the delivery of FVIIa to the surface of activated platelets to restore hemostasis when platelets are activated following vessel wall injury (Fig. 1b). As the TLT-1 receptor is absent on the surface of resting platelets and is redistributed to the surface from α-granules upon platelet aggregation^38^, HMB-001-mediated localization of FVIIa is strongly dependent on the activation status of platelets. Therefore, HMB-001, both free and FVIIa bound, will circulate latently in the blood and exert its hemostatic effect only upon vascular injury with subsequent platelet activation and TLT-1 expression. It is worth noting that other receptors, such as platelet GPIbα and endothelial protein C receptor, have been shown to interact with FVIIa, potentially influencing the localization of FVIIa-related procoagulant reactions^52^.

**Extended Data Fig. 9:**
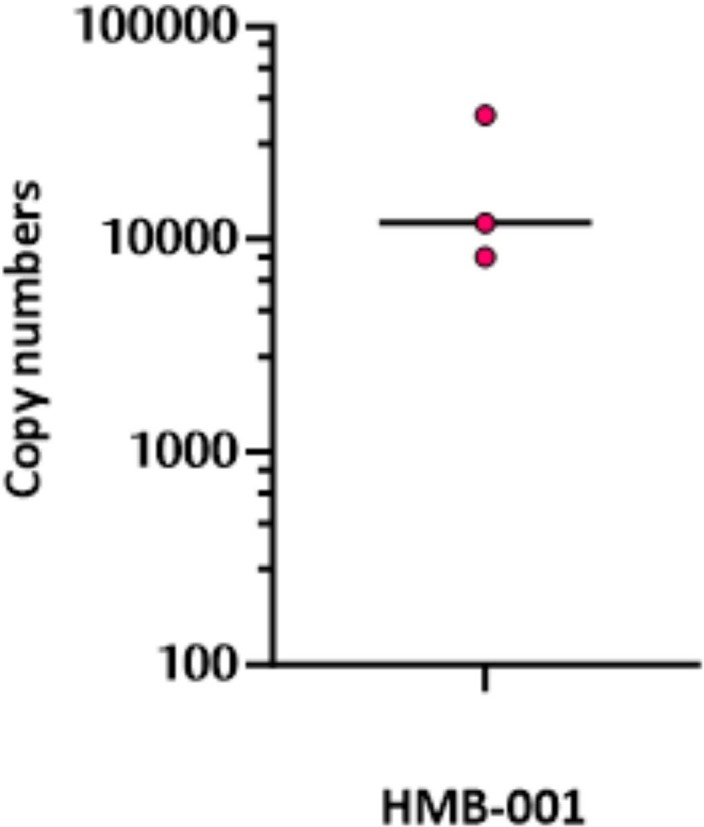
HMB-001 copy number on activated platelets. Whole blood from healthy donors (n = 3) was incubated with up to 2 µM AlexaFluor-647 labelled HMB-001 in the presence of 25 µM PAR-1 AP. HMB-001 copy number on activated platelets were calculated by conversion to arbitrary units using Quantum AlexaFluor647 MESF and Rainbow beads. Source data

Platelet aggregation is compromised in PwGT due to the lack of functional αIIbβ3 on the platelet surface (Fig. 1a)^8,53^. In the absence of effective prophylaxis, multiple intravenous administration of rFVIIa is a preferred treatment option to control episodes of acute severe bleeding. The MoA of rFVIIa involves the rescue of platelet aggregation by TF-independent fibrin generation^13,25^. One way fibrin formation on platelets might contribute to hemostasis is through fibrin-dependent pseudoaggregation. However, the exact mechanism behind this process and its contribution to hemostasis in PwGT remain to be elucidated. While αIIbβ3 is reported to have a role in the interaction between fibrin and platelets^54^, our data and others’ reports indicate that fibrin-dependent pseudoaggregation readily occurs with GT platelets^13^, suggesting that this process is independent of functional αIIbβ3. Our data show that HMB-001 potentiated FVIIa-dependent, fibrin-mediated pseudoaggregation of GT platelets (Fig. 7d) and enhanced FVIIa-dependent fibrin generation in flowing blood, achieving fibrin deposition on adhered platelets similar to that obtained with the recommended rFVIIa dose for treating ongoing bleeds. The mean (s.d.) normal plasma level of total FVII(a) is 9.1 (1.4) nM, of which FVIIa constitutes approximately 71.6 (28.8) pM (range 10–168 pM)^55,56^. As expected, our data indicate that FVII and FVIIa levels in PwGT are within the normal range. Based on clinical experiences with rFVIIa^57^ and a novel rFVIIa variant^58^, it can be estimated that a plasma FVIIa activity of >3 nM (corresponding to >6 IU ml^−1^) is needed for effective rFVIIa-based prophylactic treatment. Our data demonstrated a >3 nM rFVIIa-equivalent activity in thrombin generation assays in relevant clinical scenarios for HMB-001 (scenarios 2–5; Fig. 7g and Extended Data Fig. 7). Others have reported biological variation in TLT-1 expression on activated platelets^48,49^, and our data are in line with those observations. Differences in the copy number of TLT-1 and the strength of platelet activation^47^ will affect the number of TLT-1 receptors on the surface of activated platelets, thereby modulating the HMB-001-mediated response. Nevertheless, our data indicate that while TLT-1 expression varies among PwGT (Fig. 7c and Extended Data Fig. 9), the effects of FVIIa and HMB-001 on fibrin formation in flowing blood are robust (Fig. 8d). Through the accumulation and localization of endogenous FVIIa, HMB-001 provides an rFVIIa-equivalent activity in the hemostatically active range comparable to that obtained with therapeutic doses of exogenous rFVIIa. Combined with PK/PD modeling, these data suggest that HMB-001 could offer a subcutaneous-based prophylactic treatment with a low-frequency dosing regimen for PwGT.

HMB-001 binds equally well to FVII and FVIIa, providing a rationale for the accumulation of total FVII(a) observed in cynomolgus monkeys. Despite similar affinities, the fold accumulation of total FVII(a) was lower than that of FVIIa, likely explained by the continuous conversion of FVII to FVIIa by known physiological mechanisms involving FXa, FIXa and FVIIa itself. Upon platelet activation, it is expected that the HMB-001:FVII complex will also bind to expressed TLT-1 receptors on activated platelets. Our data confirm the potentiating effect of HMB-001 at predicted levels of FVII and FVIIa during HMB-001 treatment. Whether localized HMB-001:FVII is activated on the surface of activated platelets and thus contributes to the overall observed hemostatic activity of HMB-001 remains to be determined. In the presence of high concentrations of HMB-001, we observed a modest decline in the effect of HMB-001 in FX activation and thrombin generation assays, likely due to the competition between free HMB-001 and the HMB-001:FVIIa complex for binding to TLT-1 receptors and thus naturally dampening the effect of high-dose HMB-001 (Figs. 2c and 7g).

rFVIIa is licensed for the treatment of bleeds in people with congenital HA or HB with inhibitors. While GT and HA differ in the cause and nature of the hemostatic defect, restoration of hemostasis by pharmacological doses of rFVIIa relies on the same underlying mechanism^27^. The presented data from thrombin generation assays suggest that HMB-001 contributes sufficient hemostatic activity to support prophylaxis in HA. Further support for the utility of HMB-001 in HA comes from in vivo studies in HA mice, in which a 15-fold potency enhancement of FVIIa activity was observed when FVIIa was coformulated with an HMB-001 analogue (Fig. 2b). Thus, HMB-001 could provide an alternative prophylactic treatment option for people with HA. People with Bernard–Soulier syndrome represent another population without a prophylactic treatment option available that might benefit from HMB-001. With a defect in the platelet GPIb–V–IX receptor complex, people with Bernard–Soulier syndrome are expected to have normal FVIIa and TLT-1 levels, and treatment with rFVIIa has been shown effective^4,59^. Further investigations are needed to determine whether HMB-001 could benefit patients with FVII deficiency or HB who are known to have low levels of endogenous FVIIa^60^.

In conclusion, the present data suggest that the long half-life of HMB-001, combined with its ability to accumulate and potentiate the activity of endogenous FVIIa selectively on activated platelets, provides a sustained and localized procoagulant activity that may support prophylactic treatment of GT or other bleeding disorders for which rFVIIa has been shown efficacious in treating acute bleeds. Whether and how prophylaxis with HMB-001 would affect the treatment of breakthrough bleeds remains to be determined. In addition, the MoA of HMB-001 provides a general strategy for developing future antibody-based therapeutics using the principle of accumulation of target endogenous protein to therapeutically effective levels. An additional layer of enhancement and control of therapeutic activity can be engineered by site-specific targeting, as done with TLT-1 in the present study.

## Methods

### Inclusion and ethics

The mouse in vivo study was conducted at Novo Nordisk, Denmark. The study was approved by the Danish Animal Experiments Council, the Danish Ministry of Environment and Food, and the Novo Nordisk Welfare Body. The nonhuman primate PK/PD study was conducted at Labcorp, UK. Labcorp is licensed by the UK Home Office to undertake animal studies. The nonhuman primate PK/PD study (no. 8469444) was approved by the Labcorp Animal Welfare and Ethics Review Board. Human studies were approved by the NedMec medical ethics review board at University Medical Center Utrecht (registration no. NL5878 in the Dutch trial registry (ClinicalTrialregister.nl)), and all participants provided informed consent.

### PwGT and healthy controls

Thirteen PwGT were included in the Thrombocytopathy in the Netherlands (TiN) study, a nationwide cross-sectional study on disease phenotyping, diagnostics and genetics in people with a (suspected) platelet disorder in the Netherlands, at the University Medical Center Utrecht. PwGT were aged 30–74 years, of whom five were men and eight were women. Stored plasma samples (*n* = 13) were used for the analysis of FVII activity and FVIIa levels, and fresh blood samples (*n* = 4) were used for platelet phenotyping and functional characterization of HMB-001. Full details of the TiN study have been described elsewhere^61,62^.

Healthy controls were recruited among personnel and students at University Medical Center Utrecht by the Mini Donor biobank facility of the University Medical Center Utrecht (biobank no. 18-774). Controls were aged 24–65 years, of whom 40% were men and 60% were women. All participants provided written informed consent.

In all participants, blood was collected from the antecubital vein into trisodium citrate or sodium heparin Vacutainer tubes (BD) through phlebotomy. Blood was processed within 1–6 h of collection. Plasma was obtained within 4 h of collection by centrifugation of whole blood at 3,000*g* for 5 min. Plasma was stored at −80 °C until used.

### Preparation of recombinant proteins

FVIIa or rFVIIa (NovoSeven) was from Novo Nordisk. Recombinant zymogen FVII was produced in Chinese hamster ovary (CHO) cells and purified as described elsewhere^63^. Following purification, FVII was dialyzed into 10 mM MES, 100 mM NaCl, 10 mM CaCl_2_, pH 6.0 buffer. The level of FVIIa in the preparation was determined by measuring activity against a chromogenic substrate. By relating this to a standard curve prepared with known concentrations of rFVIIa, the measured activity could be converted to a molar concentration of FVIIa in the FVII preparation. A recombinant zymogen FVII variant (FVII-S195A) with an alanine substitution of the active site serine (S195A) was produced in CHO cells (CHO-EBNALT85, Icosagen) and purified as described elsewhere^64^. Following purification, FVII-S195A was dialyzed into 20 mM HEPES, 150 mM NaCl, 5 mM CaCl_2_, pH 7.5 buffer. The introduction of the S195A mutation was verified by the lack of activity against the chromogenic substrate. rFVIIa without the N-terminal Gla domain (FVIIa-desGLA) was produced in CHO cells (CHO-EBNA, Icosagen), purified by affinity chromatography (VIISelect, Cytiva) and desalted using a HiPrep column (Sephadex G-25, Cytiva). Active site-inhibited FVIIa (FVIIai) was prepared by incubating FVIIa-desGLA with an active site inhibitor (H-d-Phe-Phe-Arg-chloromethyl ketone (FFR-cmk), Bachem). The anti-FVIIa Fab fragment of HMB-001, used for x-ray crystallography studies, was expressed using transient transfection of HEK293 suspension cells (293Expi, Invitrogen) and purified by affinity chromatography (MabSelect SuRe, Cytiva). The anti-TLT-1 Fab fragment was produced in CHO cells (Gibco) and purified by affinity chromatography (KappaSelect, Cytiva). HMB-001 and biAb0097 were generated using the DuoBody technology^42^ by assembling the two parental monospecific bivalent antibodies, that is, the anti-FVIIa antibody with DuoBody mutations F405L and R409K and the anti-TLT-1 (pTLT-1) antibody without DuoBody mutations. HMB-001 was purified from residual levels of parental monospecific bivalent antibodies using ion exchange chromatography. The parental affinity variants of the monospecific bivalent anti-FVIIa (with DuoBody mutations F405L and R409K) and anti-TLT-1 antibodies were produced using transient transfection of HEK293 suspension cells (293Expi, Invitrogen) and purified by protein A affinity followed by size-exclusion chromatography (SEC). The affinity matrix biAbs (approximate molecular weight 150 kDa) were assembled using the DuoBody technology, as described elsewhere^42^. The two monovalent cAbs (approximate molecular weight 100 kDa) were also generated using the DuoBody technology by combining parental antibodies with an empty Fc (E216–G446 in EU numbering). The two monovalent cAbs were purified using ion exchange chromatography, and their molecular weights were confirmed using SDS–PAGE. In addition, binding between the two cAbs and their respective antigens (FVIIa or sTLT-1) was confirmed using a surface plasmon resonance (SPR) binding assay. Fragments of TF corresponding to amino acids 1–219 (sTF) and 1–244 (TF) were produced in *Escherichia coli* as described elsewhere^65,66^. Preparations of 25:75 phosphatidylserine/phosphatidylcholine vesicles with and without incorporation of TF were prepared as described elsewhere^66^. sTLT-1, corresponding to amino acids 1–147 of mature TLT-1, was expressed with a C-terminal histidine (His) tag and a two amino acid (valine–aspartic acid) linker in between using transient transfection of CHO cells (ExpiCHO, Gibco). It was then purified using the standard His-tag purification strategy (HisTrap column, Cytiva), followed by dialysis in 20 mM HEPES, 100 mM NaCl, pH 7.3. The 37-mer TLT-1 peptide, EEEEETHKIGSLAENAFSDPAGSANPLEPSQDEKSIP, corresponding to amino acids 111–147 of mature TLT-1, was prepared by standard peptide synthesis methods and is referred to as the sTLT-1 stalk peptide. Bivalent and specific heavy-chain variable domain (V_H_H) antibodies against human FVIIa^67^ and monovalent V_H_H antibodies against P-selectin (clone B10.6), fibrinogen (clone C3), GPIbα (clone 17) and fibrin (clone B12), as well as isotype control V_H_H antibody (clone R2) were produced with a tobacco etch virus (TEV) protease-cleavable N-terminal His tag in *E. coli*. V_H_H antibodies were purified from bacterial lysate with immobilized metal affinity chromatography on TALON Sepharose. His tags were removed from clones B10.6, C3, 17, R2 and B12 with TEV protease. Incompletely cleaved V_H_H, TEV protease and free His tags were removed with immobilized metal affinity chromatography. V_H_H antibodies were separated from the remaining impurities with SEC. Reactive thiols were introduced in V_H_H antibody clone 17 with Pierce *N*-succinimidyl *S*-acetylthioacetate (Thermo Fisher Scientific), followed by labeling with maleimide-activated phycoerythrin (PE) (AnaSpec) according to the manufacturer’s instructions. The anti-FVIIa V_H_H antibody and clones B10.6 and R2 were labeled with NHS-Alexa Fluor 647 (Thermo Fisher Scientific), and clones C3, R2 and B12 were labeled with NHS-Alexa Fluor 488 (Thermo Fisher Scientific) according to the manufacturer’s instructions. All reported replicate measurements were taken from distinct samples.

### Effect of an HMB-001 analogue and FVIIa on bleeding in an HA mouse model

For this in vivo study, an analogue of HMB-001 was used such that the anti-TLT-1 arm is identical to the one in HMB-001. Meanwhile, the anti-FVIIa arm of the analogue is the murine version of the corresponding arm in HMB-001 before humanization. This biAb is referred to as biAb0097. The potency of FVIIa alone and FVIIa coformulated with biAb0097 in a 1:1 molar ratio was compared in a TVT model^68,69^ in HA (*F8*^−/−^) mice expressing the human TLT-1 receptor. The mouse model was generated by replacing the mouse *treml1* gene with the human variant and breeding them with *F8*^−/−^ mice, which are exon 16‐disrupted C57Bl/129S mice. Human TLT‐1-positive *F8*^−/−^ mice were identified by PCR; the generation of this model has been described in more detail elsewhere^41^. Mice were housed under standard conditions at Novo Nordisk, Måløv, Denmark (20–23 °C, 30–60% relative humidity, a 12-h light/dark cycle, and free access to food and water) in environmentally enriched cages. The sample size was determined based on earlier similar studies in *F8*^−/−^ mice^69^. For the experiment, mice of both sexes (approximately 50:50) and 12–16 weeks of age were anesthetized with isoflurane and placed on a heating pad set to maintain animal body temperature and with the tail submerged in saline (37 °C). For practical reasons, the in vivo study with FVIIa was performed just before the study wherein FVIIa was coformulated with biAb0097. Within each study, mice were randomized and blinded. Test compounds were administered intravenously (5 ml kg^−1^) into the right lateral tail vein 5 min before the injury, as summarized in Supplementary Table 1. After transection of the left lateral vein where the tail diameter equals 2.5 mm, the bleeding time and total blood loss were recorded in a 40-min observation window as described previously^68^. If the bleeding stopped at 10, 20 or 30 min, the tail was removed from the saline and the wound was gently wiped with a saline-wetted gauze swab. Total blood loss was determined by quantifying the amount of hemoglobin (Hb) in the saline and is expressed as nmol Hb.

At the end of the observation window, a blood sample was collected from the orbital plexus into 3.8% trisodium citrate. The blood was centrifuged, plasma aliquoted and stored at −80 °C before measuring the plasma concentration of FVIIa using a luminescent oxygen channeling immunoassay. In this assay, acceptor beads were coated with an anti-FVIIa antibody (4F9, Novo Nordisk) and donor beads were coated with streptavidin; streptavidin donor beads were used in combination with a biotinylated anti-FVIIa antibody (4F7, Novo Nordisk). The FVIIa concentrations in plasma samples were determined by comparing the results to a calibration curve prepared with known amounts of human FVIIa.

Dose–response curves were generated from the measured blood loss as a function of dose or the measured plasma concentration of FVIIa at the end of the observation window. From the latter, EC_50_ values were estimated by nonlinear least-squares fitting using a three-parameter sigmoidal dose–response model (GraphPad Prism), with top and bottom values shared between the two datasets.

### Affinity of FVIIa and sTLT-1 to HMB-001 and its variants

The binding of FVIIa or sTLT-1 to biAbs was determined by SPR technology (Biacore 8K) at 25 °C (*n* = 2). Anti-human IgG (25 μg ml^−1^) was immobilized on flow cells (FC) 1 and 2 of a CM4 sensor chip (both supplied by Cytiva) using standard amine coupling chemistry. BiAbs (0.5–4 nM) were injected at a flow rate of 10 μl min^−1^ for 2 min in FC 2 alone. Subsequently, FVIIa (between 0 and 4 nM) was injected in FC 1 and 2 at a flow rate of 30 μl min^−1^ for 400 s to allow for binding to biAbs. This was followed by a 540-s running buffer injection, allowing for dissociation from biAbs. Similarly, sTLT-1 (between 0 and 60 μM) was injected in FC 1 and 2 at a flow rate of 30 μl min^−1^ for 180 s to allow for binding to biAbs. This was followed by a 360-s running buffer injection, allowing for dissociation from biAbs. The running buffer was prepared by tenfold dilution of 10× HEPES-buffered saline (HBS) with surfactant P20 (HBS-P buffer, Cytiva) and supplemented with 1 mg ml^−1^ BSA and 5 mM CaCl_2_ to give 10 mM HEPES, 150 mM NaCl, 0.05% v/v polysorbate 20, pH 7.4, 5 mM CaCl_2_ and 1 mg ml^−1^ BSA. The running buffer was also used to dilute biAb, FVIIa and sTLT-1 samples. The chip was regenerated using the recommended regeneration buffer consisting of 3 M MgCl_2_ (supplied by Cytiva) injected at a flow rate of 30 μl min^−1^ for 30 s in FC 1 and 2. Binding data were analyzed according to a Langmuir 1:1 binding kinetics model using Biacore Insight Evaluation software supplied by the manufacturer (Cytiva). For sTLT-1 binding to biAb0095 and biAb0090, binding data were analyzed according to a Langmuir 1:1 binding steady-state model using Biacore Insight Evaluation software supplied by the manufacturer (Cytiva). This SPR binding setup is preferred when comparing FVIIa or sTLT-1 binding affinities to HMB-001 and its variants, as any potential inaccuracy in biAb concentrations does not affect the affinity ranking of biAbs.

### Affinity affects biAb-mediated stimulation of FVIIa activity

To determine the effect of affinity on biAb-mediated potentiation of FVIIa activity, several anti-FVIIa and anti-TLT-1 monoclonal antibodies (mAbs) with varying affinities for FVIIa and TLT-1, respectively, were tested in a bispecific format in an FX activation assay using lipidated TLT-1 (*n* = 3). FX activation was measured in the presence of 4 nM recombinant TLT-1 incorporated into 25:75 phosphatidylserine/phosphatidylcholine vesicles, 2.5 nM FVIIa, and biAb in a concentration series from 0 to 500 nM. Following 10-min preincubation in assay buffer (50 mM HEPES, 100 mM NaCl, 10 mM CaCl_2_, pH 7.3, 1 mg ml^−1^ BSA and 0.1% polyethylene glycol (PEG) 8000) at room temperature (RT), 150 nM plasma-derived FX was added to give a total volume of 50 µl. Activation was allowed to proceed for 20 min. The activation was then terminated by adding 25 µl of quench buffer (50 mM HEPES, 100 mM NaCl, 80 mM ethylenediaminetetraacetic acid (EDTA), pH 7.3). The generated FXa was quantified by hydrolysis of 1 mM S-2765 chromogenic substrate (Chromogenix), which was observed at 405 nm for 10 min in a SpectraMax iD3 plate reader and analyzed with SoftMax Pro software (Molecular Devices). From the slope of linear absorbance increase, the specific activities of free FVIIa (*A*_free_) and FVIIa in the presence of biAb (*A*_biAb_) were determined. The stimulatory activity of each biAb concentration was calculated as the *A*_biAb_/*A*_free_ ratio and visualized with GraphPad Prism software.

### Affinity of FVIIa and FVII to HMB-001

The binding of FVIIa and FVII to HMB-001 was determined by SPR technology (Biacore 8K) at 25 °C (*n* = 2). Anti-FVII IgG1 (50 μg ml^−1^, FVII mAb CaFVII-22, MA5-17631, Invitrogen), targeting the N-terminal Gla domain of FVII, was immobilized on FC 1 and 2 of a CM4 sensor chip (Cytiva) using standard amine coupling chemistry. FVIIa (2.5 nM) or FVII (2.5 nM) was injected at a flow rate of 10 μl min^−1^ for 2 min in FC 2 alone. Subsequently, HMB-001 (between 0 and 4 nM) was injected in FC 1 and 2 at a flow rate of 30 μl min^−1^ for 300 s to allow for binding to FVIIa or FVII. This was followed by a 540-s running buffer injection, allowing for dissociation from FVIIa or FVII. The running buffer was prepared by tenfold dilution of 10× HBS-P buffer (supplied by Cytiva) and supplemented with 1 mg ml^−1^ BSA and 5 mM CaCl_2_ to give 10 mM HEPES, 150 mM NaCl, 0.05% v/v polysorbate 20, pH 7.4, 5 mM CaCl_2_ and 1 mg ml^−1^ BSA. The running buffer was also used to dilute FVIIa, FVII and HMB-001 samples. The chip was regenerated using a regeneration buffer consisting of 10 mM HEPES, 150 mM NaCl, 0.05% v/v polysorbate 20, pH 7.4 and 50 mM EDTA injected at a flow rate of 30 μl min^−1^ for 30 s in FC 1 and 2. Binding data were analyzed according to a Langmuir 1:1 binding kinetics model using Biacore Insight Evaluation software supplied by the manufacturer (Cytiva).

### Effect of HMB-001 on FVII autoactivation

To determine the effect of HMB-001 on FVII autoactivation, activation of FVII was measured in the presence of lipidated TF, zymogen FVII, a limiting amount of FVIIa, and HMB-001 (*n* = 3). Activity measurements were performed at RT in assay buffer (50 mM HEPES, 100 mM NaCl, 10 mM CaCl_2_, 0.1% PEG 8000, 1 mg ml^−1^ BSA, pH 7.3). FVII autoactivation was measured in a 50-μl reaction volume containing 2 nM FVIIa, 145 nM FVII, and 0 or 500 nM HMB-001. The reactions were initiated at different time points and incubated for 0–60 min at RT by adding 2 nM lipidated TF. Following incubation, the amount of generated FVIIa, as deduced by a calibration curve, was quantified by adding 200 nM sTF and 1 mM S-2288 chromogenic substrate (Chromogenix), which was observed at 405 nm for 10 min in a SpectraMax iD3 plate reader, analyzed with SoftMax Pro software (Molecular Devices) and visualized with GraphPad Prism software.

### Effect of HMB-001 on TF-(in)dependent activation of FX by FVIIa

To determine the effect of HMB-001 on the TF-independent and TF-dependent proteolytic activity of FVIIa, activation of FX by FVIIa was evaluated in the absence or presence of lipidated TF (*n* = 3). Activity measurements were performed at RT in assay buffer (50 mM HEPES, 100 mM NaCl, 10 mM CaCl_2_, pH 7.3, 1 mg ml^−1^ BSA and 0.1% PEG 8000). In the absence of TF, FX activation was measured in a 50-μl reaction volume containing 20 nM FVIIa, 0 or 500 nM HMB-001, and 25 µM 25:75 phosphatidylserine/phosphatidylcholine vesicles. In the presence of TF, FX activation was measured in a 50-μl reaction volume containing 100 pM FVIIa, 0 or 500 nM HMB-001, and 2 pM lipidated TF. Reactions were initiated by adding 0–250 nM (TF-independent) or 0–50 nM (TF-dependent) human plasma-derived FX and incubated for 20 min at RT. After the incubation, reactions were quenched by adding 25 μl quench buffer (50 mM HEPES, 100 mM NaCl, 80 mM EDTA, pH 7.3), followed by 1 mM S-2765 chromogenic substrate (Chromogenix). The reactions were observed at 405 nm for 10 min in a SpectraMax iD3 plate reader. The measured slope, analyzed with SoftMax Pro software (Molecular Devices), was converted to the generated FXa using a calibration curve. Enzyme kinetic parameters were estimated by nonlinear curve fitting of the data to the Michaelis–Menten equation with GraphPad Prism software.

### Effect of HMB-001 on the inhibition of FVIIa by AT

The inhibition of FVIIa by human plasma-derived AT in the absence or presence of HMB-001 was performed under pseudo-first-order conditions using a discontinuous assay (*n* = 3). The assay was conducted at RT in 50 μl assay buffer (50 mM HEPES, 100 mM NaCl, 10 mM CaCl_2_, pH 7.3, 1 mg ml^−1^ BSA and 0.1% PEG 8000) containing 200 nM FVIIa, 12 μM low-molecular-weight heparin, and 0 or 500 nM HMB-001. Following 10-min preincubation, the reaction was initiated by adding 5 μM AT at selected time points (0–2 h). The inhibition reaction was quenched by adding 0.5 mg ml^−1^ polybrene, immediately followed by adding 200 nM sTF and 1 mM S-2288 chromogenic substrate (Chromogenix). The residual FVIIa activity was measured as described above and determined as the slope of the linear progress curves. These were subsequently fitted to a first-order exponential decay function to derive the pseudo-first-order rate constant (*k*_app_) for the reaction (GraphPad Prism). An apparent second-order rate constant (*k*_inh_) was estimated as the *k*_app_ divided by the AT concentration.

### Effect of HMB-001 on the inhibition of FVIIa by TFPI

TFPI inhibition of FX activation by the FVIIa:TF complex was conducted at RT in 50 μl assay buffer (50 mM HEPES, 100 mM NaCl, 10 mM CaCl_2_, pH 7.3, 1 mg ml^−1^ BSA and 0.1% PEG 8000) containing 100 pM FVIIa, 2 pM lipidated TF, 0–20 nM recombinant TFPI, and 0 or 500 nM HMB-001 (*n* = 3). Following 10-min preincubation, the reaction was initiated by adding 50 nM plasma-derived FX and incubated for 30 min. The inhibition reaction was quenched by adding 25 μl quench buffer (50 mM HEPES, 100 mM NaCl, 80 mM EDTA, pH 7.3), immediately followed by the addition of 1 mM S-2765 chromogenic substrate (Chromogenix). The residual FXa activity was measured as described above and determined as the slope of the linear progress curves. These were subsequently fitted to a three-parameter sigmoidal dose–response model to derive the half-maximal inhibitory concentration (IC_50_) for the inhibition reaction (GraphPad Prism).

### Influence of HMB-001 on triggering TLT-1 shedding

The shedding of TLT-1 from the surface of activated platelets was assessed using citrated whole blood from healthy donors. PRP was isolated by centrifugation at 160*g* for 15 min, and 1:10 v/v acid citrate dextrose (85 mM trisodium citrate, 71 mM citric acid, 111 mM d-glucose) was added. PRP was centrifuged at 400*g* for 15 min; the plasma was discarded; and the platelet pellet was resuspended in the same volume of HEPES Tyrode (HT) buffer (145 mM NaCl, 5 mM KCl, 0.5 mM Na_2_HPO_4_, 1 mM MgSO_4_, 10 mM HEPES, 5.55 mM d-glucose, pH 6.5) with 10 ng ml^−1^ prostacyclin. The platelet suspension was centrifuged again at 400*g* for 15 min; the supernatant was discarded; and the platelet pellet was resuspended in HT buffer (pH 7.3) to a platelet count of 200 × 10^9^ l^−1^. Rested platelets (500 µl) were added to a glass cuvette (Chrono-Log) and recalcified with 3 mM CaCl_2_. Collagen Reagens HORM suspension (4 µg ml^−1^) was added to initiate aggregation, which was monitored for 1 h at 37 °C at 900 r.p.m. in a Chrono-Log model 700 aggregometer (Kordia) with AGGRO/LINK software, in the absence or presence of 100 nM HMB-001. Samples were drawn at 0, 5 and 60 min. Further shedding after these time points was inhibited by adding 25 mM EDTA. Thereafter, samples were centrifuged for 10 min at 20,000*g*. Samples were drawn from the supernatant and the resuspended aggregate pellet and were diluted 3:1 in 3× sample buffer (15.5% glycerol, 96.8 mM Tris–HCl, pH 6.8, 0.6% SDS, 0.003% bromophenol blue), reduced with 25 mM dithiothreitol, heated at 95 °C for 10 min and loaded on a 4–12% Bis–Tris precast gel. Western blot analysis was performed with 1 µg ml^−1^ polyclonal goat anti-human sTLT-1 antibody (R&D Systems) and IRDye 800CW-labeled donkey anti-goat IgG secondary antibody (LI-COR) at a 1:10,000 dilution. Data were analyzed with Empiria Studio software (LI-COR).

### Influence of HMB-001 on fibrinogen binding to sTLT-1

The influence of HMB-001 and parental monospecific bivalent anti-TLT-1 (pTLT-1) antibody on fibrinogen binding to sTLT-1 was probed by SPR technology (Biacore 8K) at 25 °C (*n* = 2) using the ABA injection strategy. Anti-His antibody (50 μg ml^−1^; His capture kit, Cytiva) was immobilized on FC 1 and 2 of a CM5 sensor chip (Cytiva) using standard amine coupling chemistry. His-tagged sTLT-1 (400 nM) was injected at a flow rate of 10 μl min^−1^ for 2 min in FC 2 alone. For the ABA strategy, solution A consisted of running buffer or 1 μM HMB-001 or 1 μM pTLT-1 antibody. Corresponding to solution A, solution B consisted of a fibrinogen concentration series (0, 62.5, 125, 250, 500 and 1,000 nM) in the presence of either running buffer or 1 μM HMB-001 or 1 μM pTLT-1 antibody (Fig. 7d). As indicated by the black horizontal lines in Fig. 7a, the preanalyte contact time was 120 s (solution A), the analyte contact time was 90 s (solution B) and the postanalyte contact time was 90 s (solution A). The flow rate was set to 30 μl min^−1^, and the ABA strategy was implemented on both FC 1 and 2. The running buffer consisted of 10 mM HEPES, 150 mM NaCl, 0.05% v/v polysorbate 20, pH 7.4, 5 mM CaCl_2_ and 1 mg ml^−1^ BSA. The running buffer was also used to dilute sTLT-1, fibrinogen and antibody samples. The chip was regenerated with a 30-s pulse of 10 mM glycine (pH 1.5) in FC 1 and 2. In addition to reference subtraction (FC 2 − FC 1), the sensorgram corresponding to 0 nM fibrinogen concentration was used as a control sensorgram and subtracted from the rest of the sensorgrams. Final binding sensorgrams were visualized using Biacore Insight Evaluation software supplied by the manufacturer (Cytiva). Fibrinogen is a homodimeric molecule and is expected to have two distinct binding sites for sTLT-1. Considering the assay setup, the binding of sTLT-1 at the first site of fibrinogen will potentially influence the binding at the second site. Therefore, data were not fit to a Langmuir 1:1 binding kinetics model.

### X-ray crystallography of HMB-001 in complex with FVIIa and TLT-1

The anti-FVIIa Fab fragment of HMB-001 was crystallized in complex with FVIIai and sTF using the hanging-drop method as described elsewhere^70^. Crystals of an SEC-purified Fab:FVIIai:sTF complex were grown using the sitting-drop vapor-diffusion technique at 18 °C. A protein solution of 360 nl protein complex (4.5 mg ml^−1^) in 20 mM HEPES, 150 mM NaCl and 0.1 mM CaCl_2_ (pH 7.4) was mixed with 360 nl of a precipitant solution containing 0.15 M CsCl and 15% w/v PEG 3350 and 360 nl water and equilibrated against 80 µl precipitant solution. Crystals grew within 6 weeks. The crystals were cryoprotected in a solution of 0.15 M CsCl, 15% w/v PEG 3350 and 20% v/v glycerol before flash cooling in liquid nitrogen. Diffraction data were collected at 100K on a Rigaku FR-X rotating anode generator equipped with a Dectris Pilatus 1M detector. Data reduction was performed with programs from the XDS package^71^. All crystallographic calculations were performed using the PHENIX (Python-based Hierarchical ENvironment for Integrated Xtallography)^72^ suite of crystallographic programs. The structure was determined by molecular replacement using the Phaser program^73^, with the coordinates of the complex structure obtained as a search model. The search model consisted of a previously crystallized similar complex wherein the Fab was the murine version of the corresponding anti-FVIIa Fab fragment of HMB-001 before humanization (unpublished work by G. Schluckebier). The asymmetric unit contains four Fab:FVIIai:sTF complexes. Iterative cycles of manual rebuilding using Coot (Crystallographic Object-Oriented Toolkit)^74^ and PHENIX refinement^72^ yielded the final model.

The anti-TLT-1 Fab and the sTLT-1 stalk peptide were mixed at a 1:2 molar ratio in HEPES buffer consisting of 20 mM HEPES (pH 7.3) and 150 mM NaCl. The 1:1 Fab–sTLT-1 stalk peptide complex was isolated using gel filtration on a Superdex 200 column eluted with HEPES buffer. It was subsequently concentrated to approximately 11 mg ml^−1^ and used for crystallization. Crystals of the gel-filtrated 1:1 molar Fab–sTLT-1 stalk peptide complex were grown using the sitting-drop vapor-diffusion technique at 18 °C. A 150-nl protein solution of 10.8 mg ml^−1^ Fab–sTLT-1 stalk peptide complex in HEPES buffer was mixed with 50 nl of 1 M LiCl, 0.1 M Na citrate–citric acid (pH 4.0) and 20% w/v PEG 6000 as precipitant and incubated over 60 µl precipitant. The crystal was cryoprotected by adding 1 µl of precipitant (20% ethylene glycol in the precipitant solution) to the crystallization drop before flash cooling in liquid nitrogen. Diffraction data were collected at 100K on the BioMAX beamline at the MAX IV synchrotron facility (Lund, Sweden) using an Eiger 16M hybrid-pixel detector from Dectris. Autoindexing, integration and scaling of the data were performed with programs from the XDS package^71^. The asymmetric unit contains two Fab–peptide complexes, as judged by Matthews coefficient analysis. The structure was determined by molecular replacement. Phaser^73^, as implemented in the program suite PHENIX^72^, was used with the chains H and L of PDB entry 5KMV as a search model localizing two Fabs. These were model-built with the correct amino acid sequence using the software Coot^74^ and thereafter refined using PHENIX refinement^75^. Amino acids 7–21 from the peptide were clearly seen in the difference electron density maps and could be model-built manually using Coot. The model was further refined using the steps of PHENIX refinement and manual rebuilding in Coot.

### Molecular model of the ternary complex on the membrane

To obtain a structure template for model building of HMB-001, we used its sequence to search the Research Collaboratory for Structural Bioinformatics PDB database for structures with homologous sequences. A structure with highly homologous sequences (PDB 5DK3)^76^ was identified. Its C-terminal 338 amino acids (out of 448) of the heavy chains and its C-terminal 110 amino acids (out of 218) of the light chains are identical to those of HMB-001. Essentially, the sequences differ only in the heavy- and light-chain hypervariable regions. The two x-ray crystallographic structures of FVIIai:sTF (PDB 8CN9) and the TLT-1 fragment (131–146) (PDB 8CHE) in complex with the two Fabs of HMB-001 described above were then applied in the following manner. The Fabs binding to FVIIai:sTF and sTLT-1(131–146), denoted anti-FVIIa Fab and anti-TLT-1 Fab, respectively, were structurally aligned using PyMOL (PyMOL molecular graphics system version 2.0, Schödinger) to those of 5DK3. Hence, in the resulting HMB-001 model, the heavy chain binding to FVIIa consists of anti-FVIIa Fab (HC(1–115)) in combination with 5DK3 (HC(113–444)), whereas the heavy chain of the other arm binding to TLT-1 consists of anti-TLT-1 Fab (HC(1–206)) combined with 5DK3 (HC(213–444)). The light chain binding to FVIIa consists of anti-FVIIa Fab (LC(1–106)) and 5DK3 (LC(111–218)) and that to TLT-1 consists of anti-TLT-1 Fab(LC). Note that the resulting HMB-001 model preserves binding to the antigens FVIIa and TLT-1 fragment (131–146). The sequence numbering was updated according to the HMB-001 sequence. As only a fraction of TLT-1 is cocrystalized with the Fab domain, its structured N-terminal domain (PDB 2FRG (TLT-1(20–125))) was combined with TLT-1(131–146) from the anti-TLT-1 Fab structure (part of the HMB-001 model), followed by an unstructured C-terminal region (residues 147–220) that harbors the transmembrane helix (residues 165–190). This was obtained using DaReUS-Loop^77,78^. Finally, models of FVIIa (in complex with TF) embedded in the membrane were obtained from Y.Z. Ohkubo^79^. Hence, aligning the FVIIa structure bound to HMB-001 with membrane-bound FVIIa results in an initial model of HMB-001 in complex with FVIIa and TLT-1(20–220) on the membrane surface.

### HMB-001 PK and PD evaluation in cynomolgus monkeys

#### Cynomolgus monkey PK study

To assess the PK properties of HMB-001 and probe its ability to accumulate endogenous FVIIa, a measure of HMB-001 PD activity, we carried out a study in cynomolgus monkeys at Labcorp (Labcorp Early Development Laboratories, Harrogate, UK). The monkeys were exposed to 12-h light/dark cycles. The ambient temperature was maintained at 19–23 °C, and the relative humidity was set at 36–77%. Three groups were administered HMB-001 subcutaneously once weekly with clinically relevant loading and maintenance doses of 1 and 0.15, 1 and 0.45, and 3 and 1.35 mg kg^−1^, respectively. A fourth group was administered an intravenous bolus injection of 3 mg kg^−1^ HMB-001. The sample size was sufficient to provide meaningful results; four naive cynomolgus monkeys (two females and two males) were included in each group. At the start of the study, the age of all animals ranged from 99 to 143 weeks, and their weight was between 2.21 and 4.46 kg.

#### Bioanalysis assays

Bioanalysis assays were used to determine the concentrations of HMB-001, total FVII(a) and FVIIa in plasma samples from cynomolgus monkeys. Values reported in Fig. 6a represent plasma concentrations for individual animals that were negative for antidrug antibody (ADA). The presence of ADAs toward the anti-FVIIa arm or the anti-TLT-1 arm was established with two separate ADA assays (not described below).

#### HMB-001 assay

For quantification of total HMB-001 in 3.2% citrated plasma from cynomolgus monkeys, an ELISA was developed and validated. To capture HMB-001, we coated an anti-idiotypic mAb against the anti-TLT-1 arm of HMB-001 (Sanquin, clone 7D11) on a microtiter plate. Samples, calibrators and quality controls (QCs) were added to the wells and incubated. After washing off unbound drug, bound HMB-001 was detected using a biotinylated anti-idiotypic mAb against the anti-FVII arm of HMB-001 (Sanquin, clone 1A9), followed by adding horseradish peroxidase (HRP)-labeled streptavidin (Sigma) and the chromogenic substrate 3,3′-5,5′-tetramethyl-benzidine (TMB) (Thermo Fisher Scientific). Substrate conversion was stopped after adding 0.2 M HCl (Sulpeco), after which the ELISA plate was analyzed on a microtiter plate reader (Epoch 2 Biotek) at 450/540 nm. During the detection step, the plate was incubated for 2 h at 37 °C, enhancing the FVII(a) target tolerance and the detection of both free and FVII(a)-bound HMB-001.

Quantification of HMB-001 was achieved by back-calculating the sample response from the HMB-001 calibration curve fitted using a logistic regression model. The assay has been validated using QC samples representing the lower limit of quantification (LLOQ, 2.70 nM), low (6.92 nM), middle (20.7 nM), high (138 nM), upper limit of quantification (ULOQ, 173 nM) and very high (8.65 μM) HMB-001 concentrations. The assay validation study showed dilution linearity up to an HMB-001 concentration of at least 8.65 μM.

#### Total FVII(a) assay

For quantification of total FVII(a) in 3.2% citrated plasma from cynomolgus monkeys, a human anti-FVII/FVIIa Asserachrom VII:Ag ELISA kit (Stago) was established, optimized to overcome drug interference and validated. The ELISA kit uses assay strips precoated with rabbit anti-human FVII/FVIIa F(ab′)2 fragment polyclonal antibody (pAb) to capture total FVII(a). Calibrators, samples (1:168) and QCs (1:168) were prepared as described below and incubated for 1 h at RT. After washing off unbound FVII(a), bound FVII(a) was detected using a rabbit anti-human FVII(a) antibody coupled to peroxidase and the chromogenic substrate TMB. Substrate conversion was stopped after adding 50 µl of 2.0 M H_2_SO_4_, after which the ELISA plate was analyzed for 15–60 min on a Biotek Epoch 2 microtiter plate reader at 450/540 nm.

Because HMB-001 binds to FVIIa and zymogen FVII, drug interference was observed in the assay setup. As such, the assay methodology was adjusted by first saturating the calibrator, samples and QCs with HMB-001 before analysis on the ELISA plate. The calibrator consisted of lyophilized human plasma with a known quantity of human FVII. Samples and QCs were prepared by mixing 3.2% citrated cynomolgus monkey plasma, a normal human plasma pool with a known quantity of total FVII(a) (internal reference pool of Sanquin), and reconstituted human plasma control with a known quantity of FVII from the assay kit with assay buffer to a 168-fold dilution. The total FVII(a) concentration was back-calculated from the calibration curve using logit regression. A run was accepted if the goodness of fit of the calibrator was ≥0.99 and if both control samples were within the reference range and deviated ≤20% when compared to the average. Samples were accepted when the deviation from the average was ≤20%. The LLOQ was 46.6% for samples in a 168-fold dilution. The ULOQ was set as the highest point of the calibrator, which equals a theoretical value of 1,616% when using a 168-fold dilution.

In the current setup, cynomolgus total FVII(a) was measured (in percentage units) using a kit for measuring human total FVII(a). Thus, the data represent human-equivalent total FVII(a) levels in cynomolgus plasma. In addition, as the calibration curve is composed of human FVII(a) in plasma instead of cynomolgus FVII(a), the obtained results are semiquantitative. The results from this assay could be used for PK modeling by converting the semiquantitative total FVII(a) levels obtained in percentages (%) to nM values. To do so, we analyzed samples containing either native human FVII or human rFVIIa against the calibration curve. This resulted in an approximate conversion factor of 16% total FVII(a) per 1 nM of total FVII(a), which was used throughout this study for reference purposes.

#### FVIIa activity assay

FVIIa activity in 3.2% citrated plasma from cynomolgus monkeys was quantified using a Staclot VIIa–rTF kit (Stago). For quantification of FVIIa activity in 3.2% citrated plasma from cynomolgus monkeys, the assay procedure was modified and fit-for-purpose qualified. The activity assay was performed on a Sysmex CS-5100 turbidimeter (Siemens Healthineers). The Sysmex turbidimeter measures transmitted light, which was converted to clotting time. The calibrator (reagent 4) containing a known quantity of human rFVIIa was dissolved in water (Fresenius Kabi). At the same time, recombinant sTF (rsTF)–phospholipids (reagent 3) and FVII-deficient plasma (reagent 2) were dissolved in water (Fresenius Kabi). First, a calibration curve was measured by placing the reagents in the Sysmex CS-5100 and the calibrator solution. A six-point calibrator was determined by measuring 1:10, 1:20, 1:40, 1:80, 1:160 and 1:320 dilutions in the Sysmex CS-5100. Before adding FVII-deficient human plasma, a prerinse with Clean 1 (1.0% sodium hypochlorite solution, Honeywell Fluka) was performed, followed by a postrinse with Clean 1. Then, the rsTF–phospholipid solution was added 20 s after the addition of FVII-deficient plasma. At 220 s after adding rsTF–phospholipids, 0.025 M CaCl_2_ (Stago) was added to induce clot formation after a prerinse with Clean 1.

After successful calibration, 3.2% citrated cynomolgus monkey plasma samples from HMB-001-treated animals were measured in a 20-fold predilution in reagent buffer. Pretreatment samples were measured undiluted. To prevent FVIIa potentiation by HMB-001, we mixed the samples with sTLT-1 peptide (Schafer-N). Before sample analysis, control samples (reagents 5a and 5b) with known concentrations of human rFVIIa were dissolved in water (Fresenius Kabi) and mixed. Samples and controls were then analyzed in three dilutions of 1:10, 1:20 and 1:40 using the same mixing sequence in the Sysmex CS-5100 as the calibrator.

Samples and controls were compared to the calibration curve using parallel-line assay analysis. For the run to be accepted, the controls had to fall within the documented reference range of the kit manufacturer, and the parallelism ratio of the controls had to fall within 0.8–1.2. In addition, samples were accepted if their parallelism ratio was within 0.8–1.2 and if at least one of the sample points was within the calibrated assay range. The LLOQ was determined at 40.5 mIU ml^−1^, and the ULOQ was theoretically set at 7,026 mIU ml^−1^.

In the current setup, cynomolgus FVIIa was measured (in mIU) using a kit for measuring human FVIIa. Thus, the data represent human-equivalent FVIIa levels in cynomolgus plasma. The equivalence between the mIU and ng units has been established, verified experimentally and found to be approximately 30 mIU ng^−1^ (ref. ^80^). This allows for further conversion of the FVIIa signals to nM, resulting in an assay range of 0.027–18.8 nM.

#### PK/PD model

PK/PD analysis of the HMB-001, FVIIa and total FVII(a) concentrations obtained in the study was carried out using a validated installation of NONMEM (version 7.4.3, ICON Development Solutions) under Windows 10 Professional and the GNU gfortran compiler version 4.5.0. Postprocessing of NONMEM analysis results was carried out in R version 4.0.5 (Comprehensive R Network (R Development Core Team, 2008)^81^). NONMEM run execution and visual predictive check were carried out using PsN (Perl-speaks-NONMEM, version 4.8.1)^82^. Parameter estimation was carried out using first-order conditional estimation with interaction.

During model development, a single additional parameter in the structural model was included if the difference in the objective function value between two models was >6.635, significant at the *P* < 0.01 level. The final model was determined based on maximized likelihood (lowest stable objective function value, physiological plausibility of parameter values, successful numerical convergence, parameter precision and acceptable visual predictive check).

The PK properties of HMB-001 were described by a two-compartment model with first-order subcutaneous absorption. FVIIa and total FVII(a) concentrations over time were modeled using turnover models in which increases in FVIIa and total FVII(a) after HMB-001 administration were described by two separate *E*_max_ functions stimulating the endogenous production of FVIIa and total FVII(a), respectively.

The developed PK/PD model was scaled to humans using established allometric scaling approaches, assuming a human body weight of 70 kg and a cynomolgus monkey weight of 2.76 kg. All clearances were allometrically scaled with a power of 0.85; volumes were scaled with a power of 1; and the absorption rate constant was scaled with a power of −0.25 (ref. ^83^). *E*_max_ was allometrically scaled with a power of 0.75 (ref. ^84^). Human baseline values of 0.067 nM (ref. ^56^) and 10 nM (ref. ^85^) were used for FVIIa and total FVII(a), respectively. The remaining parameters (Hill coefficient, EC_50_) were assumed to be similar to those of monkeys.

#### Simulations of HMB-001 dosing regimens in humans

A human body weight of 70 kg, an FVIIa baseline of 0.067 nM (ref. ^56^) and an FVII baseline of 10 nM (ref. ^85^) were used for the simulations. To illustrate the impact of interindividual variability, we simulated 1,000 participants assuming the same interindividual variability as estimated for the cynomolgus monkey data. For doses of 0.34, 0.4, 0.5 and 0.6 mg kg^−1^, the 66th, 56th, 42nd and 33rd percentiles give a twofold increase above the FVIIa baseline, respectively.

#### FVII activity and FVIIa plasma levels in GT plasma

FVII activity was measured using a prothrombin time (PT)-based factor assay. Standard clotting times were determined using 25 μl normal pooled plasma from healthy donors at 10-, 20-, 40- and 80-fold dilutions in 0.9% NaCl, mixed with 25 μl FVII-depleted plasma at 37 °C for 2 min. Plasma samples from 13 PwGT were diluted 20-fold in 0.9% NaCl, and 25 μl diluted patient plasma was mixed with 25 μl FVII-depleted plasma at 37 °C for 2 min. To initiate coagulation, we added 50 μl Dade Innovin PT reagent to the plasma samples and monitored the time to clotting.

### Evaluation of HMB-001 efficacy in Glanzmann Thrombasthenia models

To further quantify FVIIa plasma levels, we performed an ELISA using a V_H_H against FVIIa^67^. A 2 μg ml^−1^ concentration of the bivalent monoclonal anti-FVIIa-specific V_H_H in coating buffer (5 mM Na_2_CO_3_, 35 mM NaHCO_3_, 3 mM NaN_3_) was coated on a 96-well plate for 30 min at RT at 600 r.p.m. and then overnight at 4 °C. The wells were emptied and blocked for 1 h at RT with blocking buffer (blocking reagent for ELISA (Roche) + 0.05% Tween 20) at 600 r.p.m. Plasma samples from 50 healthy donors and 13 PwGT were diluted eightfold in blocking buffer, added to the wells and incubated for 1 h at RT at 600 r.p.m. The wells were washed three times, added with 50 μl of the 1:4,000 diluted primary antibody (sheep anti-FVII IgG affinity-purified, Cedarlane) in blocking buffer and incubated for 1 h at RT at 600 r.p.m. Next, the wells were washed three times with washing buffer (0.05% Tween 20 in PBS) and added with 50 μl of the 1:4,000 diluted secondary antibody (rabbit anti-sheep IgG HRP-conjugated) in blocking buffer. After incubation for 1 h at RT at 600 r.p.m., the wells were washed three times and stained with 50 μl TMB for 6 min. Substrate conversion was stopped using 25 μl of 0.3 M sulfuric acid in each well. Absorbance was measured at 450 nm, and FVIIa plasma levels were determined using a calibration curve of rFVIIa.

#### Localization of FVIIa on activated platelets

Localization of FVIIa on activated platelets was evaluated by fluorescence-activated cell sorting (FACS) with whole blood collected from healthy donors into sodium heparin Vacutainer tubes (BD). Blood was supplemented with 25 nM FVIIa with 0 or 25 nM HMB-001 in combination with 0 or 400 nM sTLT-1. Five microliters of whole blood was added to 50 µl HBS FACS buffer (10 mM HEPES, 150 mM NaCl, 1 mM MgSO_4_, 5 mM KCl, pH 7.4) with 25 μM PAR-1 AP (SFLLRN, Bachem, 141923-40-2), 1 µg ml^−1^ CRP-XL (CambCol Laboratories), Alexa Fluor 647-labeled anti-FVIIa V_H_H antibody (10 μg ml^−1^), fluorescein isothiocyanate (FITC)-labeled mouse anti-P-selectin antibody (clone VI-PL44, BD Biosciences) and R-PE-labeled anti-GPIbα V_H_H antibody (15 μg ml^−1^) and incubated at 37 °C in the dark for 10 min. Samples were fixed with 500 µl fixative buffer (137 mM NaCl, 2.7 mM KCl, 1.12 mM NaH_2_PO_4_, 1.15 mM KH_2_PO_4_, 10.2 mM Na_2_HPO_4_, 4 mM EDTA, 1.11% formaldehyde, pH 6.8) and incubated in the dark at RT for 20 min. Samples were diluted 1:1 v/v with HBS buffer and analyzed on a BD FACSCanto II flow cytometer with FACSDiva software. Platelets were gated based on forward and side scatter and GPIbα expression (Supplementary Fig. 1). FVIIa binding was expressed as median fluorescence intensity (MFI).

#### HMB-001 copy number on activated platelets

HMB-001 was conjugated to Alexa Fluor 647 by incubating 17 µM HMB-001 in sodium bicarbonate buffer (100 mM in HBS) with 1:40 vol Alexa Fluor 647 succinimidyl ester for 1 h at RT, protected from light and under continuous agitation. Free fluorophore was removed by centrifugation in a Zeba Spin desalting column (7K molecular weight cutoff). Concentration and labeling degree were determined by the absorption at 280 and 650 nm.

HMB-001 copy number on activated platelets was determined using flow cytometry. Citrated whole blood from healthy human donors (*n* = 3) was incubated with AF647-conjugated HMB-001 (31.25 nM–2 µM) for 10 min at 37 °C in HBS FACS buffer containing 25 µM PAR-1 AP or buffer, R-PE-conjugated GPIbα nanobody (in-house produced) at 15 µg ml^−1^ as a platelet marker, FITC-conjugated mouse anti-human CD62P (clone VI-PL44, BD Biosciences 555523) at a 1:25 dilution as a platelet activation marker. Then, samples were fixed using fixative buffer for 20 min at RT with protection from light. The fixed samples were diluted 1:1 in FACS buffer, and MFI was measured in a BD FACSCanto II flow cytometer. Additionally, the MFIs of Quantum Alexa Fluor 647 molecules of equivalent soluble fluorochrome (MESF) beads (Bangs Laboratories) and Rainbow calibration particles (eight peaks, 3.0–3.4 µm, BioLegend) were measured according to the manufacturer’s instructions. MFI data were converted to MESF arbitrary units based on calibration with MESF beads and corrected for the number of fluorophores per HMB-001. Maximum binding was calculated with nonlinear regression assuming a 1:1 interaction (GraphPad Prism version 10.1).

#### TLT-1 and P-selectin expression and fibrinogen binding

Fresh citrated human whole blood was warmed at 37 °C for 10 min, and 5 μl was added to 50 μl HBS with fluorescent antibodies and a platelet agonist. For quantification of TLT-1 expression, the antibodies used were Alexa Fluor 647-labeled anti-P-selectin V_H_H antibody (7.5 µg ml^−1^), R-PE-labeled anti-GPIb V_H_H antibody (15 µg ml^−1^), and Alexa Fluor 488-coupled pTLT-1 antibody (30 µg ml^−1^) or Alexa Fluor 488-labeled IgG4 isotype control (ACROBiosystems, DNP-M3-1mg, 30 µg ml^−1^). For quantification of P-selectin expression and fibrinogen binding, the antibodies used were R-PE-labeled anti-GPIb V_H_H antibody (15 µg ml^−1^) and either Alexa Fluor 647-labeled anti-P-selectin V_H_H antibody (7.5 µg ml^−1^) and Alexa Fluor 488-labeled anti-fibrinogen V_H_H antibody (25 μg ml^−1^) or Alexa Fluor 488- and Alexa Fluor 647-labeled isotype control V_H_H antibody R2. The platelet agonists used were 25 µM PAR-1 AP, 250 µM PAR-4 AP AYPGKV (produced at the peptide facility of the Netherlands Cancer Institute), 60 µM adenosine diphosphate (ADP) (Sigma, 01897), 1 µg ml^−1^ CRP-XL and 5 µM U-46619 (Cayman Chemical, 16450). This was incubated for 10 min at 37 °C in the dark to allow platelet activation. Platelet activation was stopped as described for FVIIa binding to activated platelets. Samples were subjected to FACS analysis on a BD FACSCanto II flow cytometer with FACSDiva software, and platelets were gated based on forward and side scatter and GPIbα expression. Results are expressed as MFI.

#### Influence of HMB-001 on platelet aggregation

Citrated whole blood from healthy human donors was centrifuged at 160*g* for 15 min at RT without brake to obtain PRP. The remainder of the blood was centrifuged at 2,000*g* for 10 min at RT to obtain platelet-poor plasma (PPP) to serve as a blank in the aggregometer. PRP (300 μl) was added to a glass cuvette containing a magnetic stirrer in the absence and presence of 100 nM HMB-001. Aggregation was initiated by either 5 μM ADP, 5 μM epinephrine, 4 μg ml^−1^ Collagen Reagens HORM Suspension or 10 µM PAR-1 AP. Aggregation was monitored for 15 min at 37 °C at 900 r.p.m. by light transmission aggregometry.

#### HMB-001 aggregates GT and GT-like platelets through FVIIa

Aggregation of GT and GT-like (αIIbβ3-inhibited) platelets was assessed using light transmission aggregometry in a Chrono-Log model 700 (Kordia)^13,25^. To isolate platelets, we centrifuged citrated whole blood from healthy donors at 160*g* for 15 min without brake at RT. PRP was isolated, and 1:10 v/v acid citrate dextrose (85 mM trisodium citrate, 71 mM citric acid, 111 mM d-glucose) was added. PRP was centrifuged at 400*g* for 15 min without brake at RT; plasma was discarded; and the platelet pellet was resuspended in the same volume of HT buffer (145 mM NaCl, 5 mM KCl, 0.5 mM Na_2_HPO_4_, 1 mM MgSO_4_, 10 mM HEPES, 5.55 mM d-glucose, pH 6.5) with 10 ng ml^−1^ prostacyclin. The platelet suspension was centrifuged again at 400*g* for 15 min at RT. The supernatant was then discarded, and the platelet pellet was resuspended in HT buffer (pH 7.3). The platelet count was adjusted to 200 ×10^9^ l^−1^, and platelets were rested for 30 min at RT before aggregation. To obtain GT-like platelets, we added 500 µM H-d-Arg-Gly-Asp-Trp (d-RGDW) (Bachem, 4026559) to the isolated platelets; this occupies the αIIbβ3 receptor on platelets and thereby inhibits platelet aggregation. Five hundred microliters of isolated GT-like platelets was added to a cuvette containing a magnetic stirrer. A coagulation mixture containing varying concentrations of FVIIa, 10 µg ml^−1^ human plasma-derived FX (Enzyme Research Laboratories, HFX 1010), 20 ng ml^−1^ human plasma-derived prothrombin (Enzyme Research Laboratories, HP 5530AL), 0.5 mg ml^−1^ human plasma-derived fibrinogen (Enzyme Research Laboratories, FIB3 5404L) and 3 mM CaCl_2_ was added to the platelets, and aggregation was initiated with 4 µg ml^−1^ Collagen Reagens HORM Suspension. Aggregation was monitored at 37 °C at 900 r.p.m. for 1 h.

To establish the potentiation of platelet aggregation by HMB-001, we isolated platelets from PwGT and added the coagulation mixture containing 10 nM FVIIa with or without 10 nM HMB-001 to 500 µl GT platelets. Aggregation was initiated after stimulation with 4 µg ml^−1^ Collagen Reagens HORM Suspension and monitored at 37 °C at 900 r.p.m. To establish the concentration–response profile of FVIIa and FVIIa in the presence of HMB-001, we prepared the coagulation mixture consisting of different concentrations of FVIIa (0.125–10 nM) alone and in the presence of equimolar concentrations of HMB-001. With each coagulation mixture, aggregation of GT-like platelets was initiated and monitored as described herein. For each platelet aggregation experiment, the lag time (defined as the time until half-maximal aggregation was reached) was measured and plotted against the FVIIa concentration. Data were fit to a four-parameter agonist versus response (variable slope) model using nonlinear regression to derive the EC_50_ values (GraphPad Prism). To characterize TLT-1 dependency, we performed platelet aggregation experiments using GT-like platelets in the presence of 400 nM sTLT-1 to study its effect on platelet aggregation mediated by 1 nM FVIIa with and without 1 nM HMB-001. In addition, we mimicked a clinically relevant scenario (scenario 3, Supplementary Table 8) using GT-like washed platelets (*n* = 3). The predicted accumulated levels of total FVII(a) and FVIIa for clinical scenario 3 (Supplementary Table 8) were reconstituted by adding FVII-S195A and FVIIa. HMB-001 was added as indicated. Aggregation was induced with 4 µg ml^−1^ collagen in buffer with 3 mM CaCl_2_, 10 μg ml^−1^ FX, 20 ng ml^−1^ prothrombin and 0.5 mg ml^−1^ fibrinogen. Light transmission was monitored for 1 h (Fig. 7).

#### HMB-001 affects thrombin generation in GT-like platelets

The effect of HMB-001 on FVIIa in the presence of FVII was evaluated by a calibrated automated thrombin generation assay in human PRP under GT-like conditions and compared to the effect of rFVIIa alone. The PRP thrombin generation assay was made sensitive to FVIIa by introducing HA-like conditions and antagonizing any residual TF in plasma. In this context, the predicted plasma levels of HMB-001, FVIIa and total FVII(a) were reconstituted, and the effect on thrombin generation was evaluated following the initiation of platelet activation. The background plasma concentrations of FVIIa and total FVII(a) were assumed to be 67 pM (ref. ^56^) and 10 nM, respectively. The predicted accumulated levels of total FVII(a) and FVIIa for the five different clinical scenarios (Supplementary Table 8) were reconstituted by adding FVII-S195A and FVIIa after accounting for the background plasma concentration of total FVII(a) and FVIIa, respectively. Citrated human whole blood was centrifuged for 15 min at 160*g* without brake to obtain PRP. The remainder of the blood was centrifuged for 10 min at 2,000*g* to obtain PPP, which was used to adjust the platelet count to 250 × 10^9^ l^−1^. PRP was incubated for 60 min at 37 °C with 500 µM d-RGDW to mimic GT conditions, 25 µg ml^−1^ neutralizing sheep anti-FVIII pAb and 10 nM neutralizing mouse anti-TF mAb. Next, the predicted plasma levels of HMB-001, FVIIa and total FVII(a) from the five different clinical scenarios in HBS/0.2% BSA buffer were added to the PRP (as indicated in Supplementary Table 8), and platelets were activated with a trigger mixture consisting of 30 µM PAR-1 AP and 1 µg ml^−1^ CRP-XL for 10 min at 37 °C. Finally, thrombin generation was initiated by adding 16.7 mM CaCl_2_ and monitored by measuring fluorogenic thrombin substrate (Z-Gly-Gly-Arg-7‐amino‐4‐methylcoumarin·HCl (I-1140)) conversion at 400/500 nm for 3 h at 37 °C in a SpectraMax iD3 microtiter plate reader (Molecular Devices) with SoftMax Pro software. Thrombin generation was deduced from fluorescence data as previously described^86^.

#### HMB-001 affects fibrin formation in GT and GT-like platelets

Coverslips were functionalized by incubating in 1:1 v/v 12.1 N hydroxychloride and methanol for 30 min at RT, followed by incubating with a 1:100 dilution of 95% aminopropyltriethoxysilane in ethanol for 30 s at RT. Finally, coverslips were incubated in a 1:20 dilution of 25% glutaraldehyde in HBS for 1 h at RT. Washing steps in between were performed with water or ethanol. Coverslips were dried and stored in HBS at 4 °C until use. Before use, coverslips were washed^87^, dried and coated with 0.3 mg ml^−1^ collagen type I solution from bovine skin (Sigma) in HBS for 1 h, then blocked with HBS/1% BSA buffer at 4 °C overnight. Polydimethylsiloxane parallel-plate microfluidic devices were washed three times with hydrochloric acid, acetone and 96% ethanol for 15 min. Next, devices were rinsed and blocked with HBS/1% BSA buffer at 4 °C overnight. Washed and dried coverslips were attached to the polydimethylsiloxane device using vacuum and mounted on a Zeiss Axiovert Observer Z1 widefield fluorescence microscope with Colibri LEDs. Platelets were labeled with MitoTracker Orange CMTMRos (Thermo Fisher Scientific) for 30 min. Citrated human whole blood was subsequently supplemented with 8.3 μg ml^−1^ Alexa Fluor 488-conjugated V_H_H antifibrin and 20 μg ml^−1^ corn trypsin inhibitor (Enzyme Research Laboratories) with or without 0–25 nM FVIIa or 0–100 nM HMB-001 and was recalcified with 20 mM CaCl_2_. In healthy controls, αIIbβ3 was blocked with 500 μM d-RGDW, and blood was aspirated through the flow chamber with a syringe pump at a shear rate of 300 s^−1^. Platelet adhesion and fibrin deposition were monitored at 200× magnification at a frame rate of three per minute for 20 min.

### Reporting summary

Further information on research design is available in the Nature Portfolio Reporting Summary linked to this article.

### Supplementary information

Supplementary Information Supplementary Text, Tables 1–8 and Fig. 1.

Reporting Summary

### Source data

Source Data Fig. 2 Statistical source data.

Source Data Fig. 3 Statistical source data.

Source Data Fig. 4 Statistical source data.

Source Data Fig. 6 Statistical source data.

Source Data Fig. 7 Statistical source data.

Source Data Fig. 8 Statistical source data.

Source Data Extended Data Fig. 1 Statistical source data.

Source Data Extended Data Fig. 2 Statistical source data.

Source Data Extended Data Fig. 3 Statistical source data.

Source Data Extended Data Fig. 4 Statistical source data.

Source Data Extended Data Fig. 5 Statistical source data.

Source Data Extended Data Fig. 7 Statistical source data.

Source Data Extended Data Fig. 8 Statistical source data.

Source Data Extended Data Fig. 9 Statistical source data.

## Data Availability

The authors have provided all data associated with the investigations reported in this article alongside the relevant source data. The crystal structures generated in this study have been deposited in the Protein Data Bank (identifiers 8CHE and 8CN9). Source data are provided with this paper.
