# Supplementary material for: A bispecific antibody approach for the potential prophylactic treatment of inherited bleeding disorders
Source: Nat Cardiovasc Res. 2024 Feb 8;3(2):166–85. doi: 10.1038/s44161-023-00418-4 (PMC11358003; doi:10.1038/s44161-023-00418-4)

nature cardiovascular research

Article https://doi.org/10.1038/s44161-023-00418-4

A bispecific antibody approach for the potential prophylactic treatment of inherited bleeding disorders

In the format provided by the authors and unedited

Supplement

Contents page

HMB-001 analogue enhances the potency of FVIIa by about 15-fold in the tail vein transection mouse injury model.

Supplementary Table 1. Overview of treatment groups and test articles administered to transgenic haemophilia A mice expressing human TLT-1.

Supplementary Table 2. Binding characteristics of different biAb compositions to FVIIa and sTLT-1 determined with SPR at 25°C and pH 7.4.

Supplementary Table 3. Binding characteristics of HMB-001 to FVIIa, FVII and sTLT-1 determined with SPR at 25°C and pH 7.4.

Supplementary Table 4. Effect of HMB-001 on catalytic activity of FVIIa and FVIIa inhibition.

Supplementary Table 5. Data collection refinement statistics from X-ray structure determination of the complex between a) FVIIai, sTF and anti-FVIIa Fab of HMB-001 and b) stalk region of TLT-1 receptor and anti-TLT-1 Fab of HMB-001.

Supplementary Table 6. Pre-dose and C_{max} plasma levels of endogenous FVIIa and total FVII(a) (measured as human equivalent), and HMB-001 in anti-drug antibody (ADA)-negative plasma samples in cynomolgus monkeys.

Supplementary Table 7A. Mean platelet counts during the HMB-001 PK study in cynomolgus monkeys.

Supplementary Table 7B. Mean change in body weight of cynomolgus monkeys in HMB-001 PK study.

Supplementary Table 8. Predicted plasma levels of FVIIa, total FVII(a) and HMB-001 at 5 different clinical scenarios corresponding to 5 different dosing regimens.

Supplementary Figure 1. Flow cytometry gating strategy.

HMB-001 analogue enhances the potency of FVIIa by about 15-fold in the tail vein transection mouse injury model

The ability of biAb to potentiate FVIIa activity *in vivo* was evaluated in transgenic haemophilia A (HA) mice expressing human TLT-1 and using the tail vein transection (TVT) injury model. Overview of treatment groups and test articles are provided in Supplementary Table 1. For the co-administration of FVIIa and biAb0097 (FVIIa:biAb0097), the two compounds were given in equimolar (1:1) amounts.

Supplementary Table 1. Overview of treatment groups and test articles administered to transgenic haemophilia A mice expressing human TLT-1.

Test article	Dose in nmol/kg [number of animals per dose]
FVIIa	0.001 [n=10], 0.5 [n=9], 2 [n=10], 4 [n=10], 6 [n=9], 8 [n=10], 20 [n=10], 40 [n=3]
FVIIa:biAb0097	0.01:0.01 [n=6], 0.05:0.05 [n=6], 0.1:0.1 [n=6], 0.5:0.5 [n=6], 2:2 [n=6]

As shown in Extended Data Fig. 1a and 1b, administration of FVIIa resulted in dose-dependent and FVIIa plasma concentration-dependent reductions of blood loss, respectively. Upon co-formulation of FVIIa with an equimolar amount of the HMB-001 sequence analogue (biAb0097), a left-shift of the dose- and concentration-response curves were observed corresponding to an apparent increased haemostatic effect of FVIIa. To account for a shorter half-life and consequently faster *in vivo* elimination of free FVIIa compared to the FVIIa:biAb0097 complex, the effect of biAb0097 was estimated from the EC_{50}-values derived from the concentration-response profiles where the concentrations of FVIIa were measured at the end of the bleeding window. With estimated EC_{50}-values (95% confidence intervals in square brackets) of 5.4 nM [1.7–14.3] and 0.36 nM [0.005–1.3] for FVIIa and the FVIIa:biAb0097 co-formulation, respectively, it could be concluded that biAb0097 reduces the EC_{50}-value of FVIIa by approximately 15-fold. This in turn corresponds to an apparent 15-fold potency enhancement of FVIIa by biAb0097.

Supplementary Table 2. Binding characteristics of different biAb compositions to FVIIa and sTLT-1 determined with SPR at 25°C and pH 7.4.

BiAb	FVIIa binding characteristics				sTLT-1 binding characteristics			
	k_{on} (1/Ms)	k_{off} (1/s)	$K_{D,\ FVIIa}$ (nM)	Fold difference in K_D	k_{on} (1/Ms)	k_{off} (1/s)	$K_{D,\ sTLT\text{-}1}$ (nM)	Fold difference in K_D
HMB-001	2.90E+06	1.27E-04	0.04	1.0	2.74E+05	6.41E-04	2.34	1.0
biAb0011	2.72E+06	5.70E-04	0.21	4.8	3.08E+05	6.37E-04	2.07	0.9
biAb0012	1.74E+06	2.82E-03	1.63	37.1	2.96E+05	6.58E-04	2.22	1.0
biAb0013	1.88E+06	1.76E-02	9.61	219.5	2.97E+05	6.79E-04	2.29	1.0
biAb0014	1.34E+05	7.15E-02	532.5	12171.4	2.83E+05	5.82E-04	2.06	0.9
biAb0095	2.85E+06	1.21E-04	0.04	1.0	NA	NA	12.95	5.5
biAb0090	2.81E+06	1.35E-04	0.05	1.1	NA	NA	196.50	84.0

k_{on}, association rate; k_{off}, dissociation rate; K_D, equilibrium dissociation constant; NA, Not applicable.

Supplementary Table 3. Binding characteristics of HMB-001 to FVIIa, FVII and sTLT-1 determined with SPR at 25°C and pH 7.4.

HMB-001	Run	k_{on} ± SE (1/Ms)	k_{off} ± SE (1/s)	K_D (nM)	Rmax (RU)	Chi^2 (RU^2)	tc
FVIIa	Run 1	1.80E+06 ± 7.57E+02	6.66E-04 ± 5.45E-07	0.37	46.8	1.51e-01	3.58e12
	Run 2	1.80E+06 ± 7.20E+02	6.64E-04 ± 5.18E-07	0.37	44.2	1.22e-01	1.55e12
	**Average**	**1.80E+06**	**6.65E-04**	**0.37**			
FVII	Run 1	1.83E+06 ± 8.61E+02	7.25E-04 ± 6.15E-07	0.4	28.6	6.95e-02	8.66e11
	Run 2	1.82E+06 ± 8.79E+02	7.25E-04 ± 6.13E-07	0.4	27.6	6.91e-02	1.95e12
	**Average**	**1.83E+06**	**7.25E-04**	**0.4**			
sTLT-1	Run 1	2.62e+05 ± 1.93E+02	6.10e-04 ± 1.48E-06	**2.33**	11.7	2.73e-02	1.05e12
	Run 2	2.86e+05 ± 1.87E+02	6.72e-04 ± 1.32E-06	**2.35**	10.9	1.97e-02	7.18e09
	**Average**	**2.74e+05**	**6.41e-04**	**2.34**			

k_{on}, association rate; k_{off}, dissociation rate; K_D, equilibrium dissociation constant; Rmax, maximum response when all ligand is occupied; Chi^2, closeness of the fit; tc, mass transfer coefficient.

Supplementary Table 4. Effect of HMB-001 on catalytic activity of FVIIa and FVIIa inhibition.

FX activation	Without tissue factor		With tissue factor	
**Mean (SD)**	**Without HMB-001**	**With HMB-001**	**Without HMB-001**	**With HMB-001**
**k_{cat}, s^{-1}**	1.11 (0.04) × 10^{-4}	0.77 (0.04) × 10^{-4}	1.21 (0.09)	1.27 (0.10)
**K_m, nM**	43 (5)	19 (4)	10 (2)	11 (2)
**k_{cat}/K_m, nM^{-1} s^{-1}**	2.6 (0.2) × 10^{-6}	4.1 (0.6) × 10^{-6}	0.12 (0.02)	0.11 (0.02)

k_{cat}: turnover number; K_m: Michaelis constant; k_{cat}/k_m: catalytic efficiency.

FVIIa inhibition		
**Mean (SD)**	**Without HMB-001**	**With HMB-001**
**AT, k_i, $M^{-1}s^{-1}$**	82 (9)	98 (11)
**TFPI, IC_{50}, nM**	0.56 (0.1)	0.58 (0.08)

AT: antithrombin; k_i: inhibitor constant; TFPI: tissue factor pathway inhibitor; IC_{50}: concentration at which 50% of the activity of 100 pM FVIIa is inhibited.

Supplementary Table 5. Data collection refinement statistics from X-ray structure determination of the complex between a) FVIIai, sTF and anti-FVIIa Fab of HMB-001 and b) stalk region of TLT-1 receptor and anti-TLT-1 Fab of HMB-001.

a)

**Parameter**	**Result**
Wavelength (Å)	1.5418
Resolution range (Å)	48.5 – 3.4 (3.52 – 3.4)
Space group	P 21 (No. 4)
Unit cell (Å)	144.78 100.79 181.74
Unit cell (deg)	90 101.23 90
Total reflections	340557 (24056)
Unique reflections	69058 (6423)
Multiplicity	4.9 (3.7)
Completeness (%)	96.3 (91.4)
Mean I/sigma (I)	3.59 (0.86)
Wilson B-factor	63.86
R-merge	0.31 (1.05)
R-meas	0.347 (1.22)
R-pim	0.151 (0.603)
CC1/2	0.98 (0.697)
CC*	0.995 (0.906)
Reflections used in refinement	684175 (6423)
Reflections used for R-free	1957 (180)
R-work	0.3030 (0.4045)
R-free	0.3549 (0.4551)
CC (work)	0.881 (0.682)
CC (free)	0.853 (0.514)
Number of non-hydrogen atoms	29520
macromolecules	29476
ligands	44
Protein residues	3818
RMS (bonds)	0.002
RMS (angles)	0.50
Ramachandran favored (%)	95.48
Ramachandran allowed (%)	4.52
Ramachandran outliers (%)	0.00
Rotamer outliers (%)	0.84
Clashscore	7.27
Average B-factor (Å2)	64.30
macromolecules	64.27
ligands	83.61
PDB accession code	8CN9

b)

**Parameter**	**Result**
Wavelength	0.9799
Resolution range	27.19 - 1.49 (1.543 - 1.49)
Space group	P 1
Unit cell (Å, deg)	53.23 65.38 67.15 91.88 91.72 92.89
Total reflections	260182 (25651)
Unique reflections	140068 (9126)
Multiplicity	1.9 (1.9)
Completeness (%)	91.03 (62.10)
Mean I/sigma(I)	9.03 (1.37)
Wilson B-factor (Å^2)	18.72
R-merge	0.053 (0.59)
R-meas	0.074 (0.84)
R-pim	0.052 (0.59)
CC1/2	0.994 (0.0678)
CC*	0.999 (0.356)
Reflections used in refinement	134358 (9123)
Reflections used for R-free	1783 (123)
R-work	0.1560 (0.2388)
R-free	0.1734 (0.2490)
CC(work)	0.964 (0.680)
CC(free)	0.961 (0.679)
Number of non-hydrogen atoms	8036
macromolecules	7017
ligands	0
solvent	1019
Protein residues	896
RMS(bonds)	0.009
RMS(angles)	0.96
Ramachandran favored (%)	97.29
Ramachandran allowed (%)	2.60
Ramachandran outliers %)	0.11
Rotamer outliers (%)	0.50
Clashscore	2.09
Average B-factor (Å^2)	24.02
macromolecules	22.53
solvent	34.28
Number of TLS groups	1
PDB accession code	8CHE

Statistics for the highest-resolution shell are shown in parentheses. Definitions of standard abbreviations included in the table are defined[88]

Supplementary Table 6. Pre-dose and C_{max} plasma levels of endogenous FVIIa and total FVII(a) (measured as human equivalent), and HMB-001 in anti-drug antibody (ADA)-negative plasma samples in cynomolgus monkeys.

	FVIIa		Total FVII(a)		HMB-001
	Pre-dose (nM)	C_{max} (nM)	Pre-dose (nM)	C_{max} (nM)	C_{max} (nM)
**Group 1**	0.12 ± 0.04	0.9 ± 0.2	4.8 ± 0.9	14.8 ± 1.4	47 ± 4
**Group 2**	0.08 ± 0.03	0.6 ± 0.1	4.3 ± 1.5	14.3 ± 3.1	41 ± 4
**Group 3**	0.08 ± 0.03	1.6 ± 0.7	4.2 ± 0.8	23.3 ± 6.0	157 ± 20
**Group 4**	0.10 ± 0.01	1.6 ± 0.2	4.7 ± 0.2	20.0 ± 1.7	384 ± 158

Results are shown as the mean ± SD for the four animals in each group. C_{max} is the highest mean plasma level up to and including Day 6 where all animals are ADA negative. Cynomolgus monkey endogenous FVIIa and total FVII(a) were measured using modified human FVIIa clot activity and human FVII ELISA kits by Stago and hence are referred to as human equivalent (see Materials and Methods).

Supplementary Table 7A. Mean platelet counts during the HMB-001 PK study in cynomolgus monkeys.

	Pre-dose		Day 1		Day 19	
	Male (n=2)	Female (n=2)	Male (n=2)	Female (n=2)	Male (n=2)	Female (n=2)
**Group 1**	420 ± 9.9	471 ± 60.1	396 ± 33.9	442 ± 64.3	453 ± 58.7	475 ± 98.3
**Group 2**	272 ± 50.2	389 (n=1)	289 ± 41.7	411 ± 44.5	314 ± 28.3	427 ± 99
**Group 3**	402 ± 116	339 ± 48.8	422 ± 149.9	384 ± 68.6	495 ± 139.3	343 ± 38.2
**Group 4**	530 ± 63.6	422 (n=1)	482 ± 18.4	415 ± 9.2	393 ± 55.2 (Day 14)	298 ± 141.4 (Day 14)

Results are shown as the mean ± SD for the 2 animals/sex/group in each group. Platelet counts are in 10E9/L. Pre-dose measurement was day 8 before dosing was initiated. For Group 4, platelet counts were measured on Day 14 instead of Day 19.

Supplementary Table 7B. Mean change in body weight of cynomolgus monkeys in HMB-001 PK study.

	Day (1 - 8)		Day (8 - 15)		Day (15 - 22)	
	Male (n=2)	Female (n=2)	Male (n=2)	Female (n=2)	Male (n=2)	Female (n=2)
**Group 1**	0.12 ± 0.078	0.05 ± 0.014	0.07 ± 0.057	0.05 ± 0.014	0.04 ± 0.000	0.02 ± 0.021
**Group 2**	0.11 ± 0.049	0.02 ± 0.007	0.04 ± 0.021	0.06 ± 0.007	0.01 ± 0.071	0.00 ± 0.028
**Group 3**	0.09 ± 0.035	0.09 ± 0.021	-0.04 ± 0.042	0.10 ± 0.035	0.05 ± 0.014	0.02 ± 0.000
**Group 4**	0.06 ± 0.014	0.04 ± 0.000	0.01 ± 0.035	-0.03 ± 0.042	0.04 ± 0.007	0.01 ± 0.035

Results are shown as the mean ± SD for the 2 animals/sex/group in each group. Change in body weight is presented in units of kg.

Supplementary Table 8. Predicted plasma levels of FVIIa, total FVII(a) and HMB-001 at 5 different clinical scenarios corresponding to 5 different dosing regimens.

	Target FVIIa (nM)	Predicted total FVII(a) (nM)	Predicted HMB-001 (nM)	HMB-001 QW dose (mg/kg)
1	0.21	14.9	27.6	0.16
2	0.52	17.95	58.7	0.34
3	1	20.9	116	0.67
4	1.38	23	191,7	1,1
5	1.78	26.6	863	5

QW, once weekly

Supplementary Figure 1. Flow cytometry gating strategy

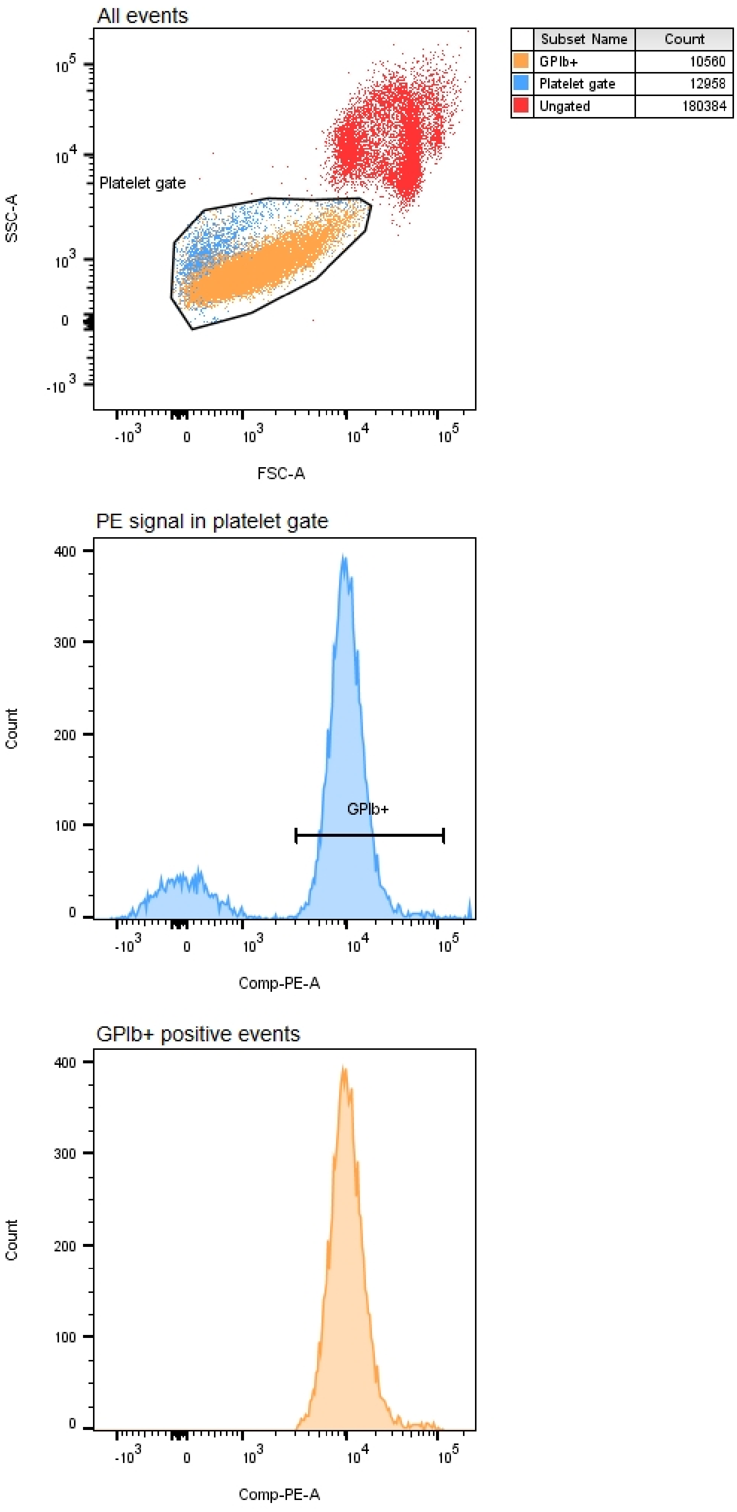

Supplement: Supplementary file 1 — Supplementary Text, Tables 1–8 and Fig. 1. [file 44161_2023_418_MOESM1_ESM.pdf]
