# Supplementary material for: A bispecific antibody approach for the potential prophylactic treatment of inherited bleeding disorders
Source: Nat Cardiovasc Res. 2024 Feb 8;3(2):166–85. doi: 10.1038/s44161-023-00418-4 (PMC11358003; doi:10.1038/s44161-023-00418-4)
Supplement: Supplementary file 2 — Reporting Summary [file 44161_2023_418_MOESM2_ESM.pdf]

nature portfolio

Corresponding author(s): Prafull S. Gandhi, Rolf T. Urbanus

Last updated by author(s): Dec 14, 2023

Reporting Summary

Nature Portfolio wishes to improve the reproducibility of the work that we publish. This form provides structure for consistency and transparency in reporting. For further information on Nature Portfolio policies, see our Editorial Policies and the Editorial Policy Checklist.

Statistics

For all statistical analyses, confirm that the following items are present in the figure legend, table legend, main text, or Methods section.

n/a	Confirmed	
☐	☒	The exact sample size (*n*) for each experimental group/condition, given as a discrete number and unit of measurement
☐	☒	A statement on whether measurements were taken from distinct samples or whether the same sample was measured repeatedly
☐	☒	The statistical test(s) used AND whether they are one- or two-sided<br>*Only common tests should be described solely by name; describe more complex techniques in the Methods section.*
☒	☐	A description of all covariates tested
☒	☐	A description of any assumptions or corrections, such as tests of normality and adjustment for multiple comparisons
☐	☒	A full description of the statistical parameters including central tendency (e.g. means) or other basic estimates (e.g. regression coefficient) AND variation (e.g. standard deviation) or associated estimates of uncertainty (e.g. confidence intervals)
☐	☒	For null hypothesis testing, the test statistic (e.g. *F*, *t*, *r*) with confidence intervals, effect sizes, degrees of freedom and *P* value noted<br>*Give P values as exact values whenever suitable.*
☒	☐	For Bayesian analysis, information on the choice of priors and Markov chain Monte Carlo settings
☒	☐	For hierarchical and complex designs, identification of the appropriate level for tests and full reporting of outcomes
☒	☐	Estimates of effect sizes (e.g. Cohen's *d*, Pearson's *r*), indicating how they were calculated

Our web collection on statistics for biologists contains articles on many of the points above.

Software and code

Policy information about availability of computer code

Data collection	SPR: Biacore Insight Control software (Cytiva), Activity assays with chromogenic substrates and ELISA: Softmax Pro (Molecular Devices) or BioTek Epoch 2 onboard Software as indicated, LTA: AGGRO/LINK software (Chrono-log): Western-blot: Emperia Studio Software (LI-COR), FACS: FACSDiva™ (BD)
Data analysis	All data analyses and statistical analyses were performed with GraphPad Prism software unless otherwise indicated; SPR: Biacore Insight Evaluation Software, Empiria Studio software (LI-COR), crystallography: XDS Package; Phenix Suite; Phaser; COOT. Modelling: The PyMOL Molecular Graphics System, Version 2.0; DaReUs loop builder, PK/PD analysis: NONMEM version 7.4.3 (ICON Development Solutions), GNU gfortran compiler version 4.0.5, Perl-speaks-Nonmem version 4.8.1, FACS: FACSDiva™, Microscopy: ZEN 2 Blue Edition.

For manuscripts utilizing custom algorithms or software that are central to the research but not yet described in published literature, software must be made available to editors and reviewers. We strongly encourage code deposition in a community repository (e.g. GitHub). See the Nature Portfolio guidelines for submitting code & software for further information.

Data

Policy information about availability of data

All manuscripts must include a data availability statement. This statement should provide the following information, where applicable:

- Accession codes, unique identifiers, or web links for publicly available datasets
- A description of any restrictions on data availability
- For clinical datasets or third party data, please ensure that the statement adheres to our policy

The authors have provided all data associated with the investigations reported in this manuscript, alongside the relevant source data.

Research involving human participants, their data, or biological material

Policy information about studies with human participants or human data. See also policy information about sex, gender (identity/presentation), and sexual orientation and race, ethnicity and racism.

Reporting on sex and gender	People with Glanzmann Thrombasthenia (n=13) were aged 30–74 years, of whom five were men, and eight were women. Healthy controls were aged 24-65 years, of whom 40% were men and 60% were women.
Reporting on race, ethnicity, or other socially relevant groupings	These data were not available
Population characteristics	People with Glanzmann Thrombasthenia (n=13) were aged 30–74 years, of whom five were men, and eight were women. Healthy controls were aged 24-65 years, of whom 40% were men and 60% were women.
Recruitment	Thirteen People with Glanzmann Thrombasthenia were included in the Thrombocytopathy in the Netherlands (TiN) study, a nationwide cross-sectional study on disease phenotyping, diagnostics, and genetics in people with a (suspected) platelet disorder in the Netherlands, at the University Medical Center Utrecht. Healthy controls were recruited amongst personnel and students at University Medical Center Utrecht by the MiniDonor biobank facility of the University Medical Center Utrecht (Biobank number 18-774). All participants provided informed consent.
Ethics oversight	Approval for the human studies was obtained from the NedMec medical ethics review board at UMC Utrecht (registration number NL5878 in Dutch trial registry (ClinicalTrialregister.nl))

Note that full information on the approval of the study protocol must also be provided in the manuscript.

Field-specific reporting

Please select the one below that is the best fit for your research. If you are not sure, read the appropriate sections before making your selection.

☒ Life sciences ☐ Behavioural & social sciences ☐ Ecological, evolutionary & environmental sciences

For a reference copy of the document with all sections, see nature.com/documents/nr-reporting-summary-flat.pdf

Life sciences study design

All studies must disclose on these points even when the disclosure is negative.

Sample size	Sample size for mouse studies were based on earlier similar studies in F8-/- mice as described by Johansen, P.B., Tranholm, M., Haaning, J., Knudsen, T. Development of a tail vein transection bleeding model in fully anaesthetized haemophilia A mice - characterization of two novel FVIII molecules. Haemophilia 22, 625-631 (2016). Sample size for PK/PD studies in Cynomolgus monkeys was sufficient to provide meaningful results (n=4).
Data exclusions	For the PK/PD study in Cynomolgus monkeys described in figure 6a, data on FVIIa concentration, HMB-001 concentration and total FVII(a) concentration were excluded for anti-drug antibody positive plasma samples.
Replication	All experiments were repeated at least twice.
Randomization	For the mice study (Fig 2B and Extended Fig 1), the in vivo study with FVIIa was run just before the study wherein FVIIa was co-formulated with biAb0097. Within each study, mice were randomized and blinded.
Blinding	For the mice study (Fig 2B and Extended Fig 1), the in vivo study with FVIIa was run just before the study wherein FVIIa was co-formulated with biAb0097. Within each study, mice were randomized and blinded.

Reporting for specific materials, systems and methods

We require information from authors about some types of materials, experimental systems and methods used in many studies. Here, indicate whether each material, system or method listed is relevant to your study. If you are not sure if a list item applies to your research, read the appropriate section before selecting a response.

Materials & experimental systems

n/a	Involved in the study
☐	☒ Antibodies
☐	☒ Eukaryotic cell lines
☒	☐ Palaeontology and archaeology
☐	☒ Animals and other organisms
☐	☒ Clinical data
☒	☐ Dual use research of concern
☒	☐ Plants

Methods

n/a	Involved in the study
☒	☐ ChIP-seq
☐	☒ Flow cytometry
☒	☐ MRI-based neuroimaging

Antibodies

Antibodies used

Bioanalysis assay to measure plasma concentration of FVIIa in HA mouse Tail Vein Transection (TVT) model using Luminescent Oxygen Channelling Immunoassay (LOCI): Acceptor beads were coated with anti-FVIIa antibody, clone 4F9 (Novo Nordisk A/S, validated for LOCI assay). Donor beads with streptavidin: biotinylated anti-FVIIa antibody, clone 4F7 (Novo Nordisk A/S, validated for LOCI assay)

Binding study using Surface Plasmon Resonance: Anti-human IgG to capture HMB-001 and its variants (Cytiva, Lot #: 10339007, Batch #: 29294600). Anti-FVII IgG1: anti-FVII mAb to capture FVIIa and FVII (Invitrogen, CaFVII-22, MA5-17631, Lot #: YB3832562, Batch #: 8008743723, RRID # AB_2539021)

Bioanalysis assays to probe HMB-001 pharmacokinetics and pharmacodynamics in cynomolgous monkeys:
HMB-001 ELISA assay: anti-idiotypic mAb against the anti-TLT-1 arm of HMB-001, clone 7D11 (Sanquin in-house produced, validated for HMB-001 ELISA). Anti-idiotypic mAb against the anti-FVII arm of HMB-001, clone 1A9 (Sanquin in-house produced, validated for HMB-001 ELISA). Total FVII(a) ELISA assay using human anti-FVII/FVIIa Asserachrom® VII:Ag ELISA kit (Stago B.V., Leiden, The Netherlands): rabbit anti-human FVII/FVIIa F(ab')2 fragments polyclonal antibody (Stago B.V., polycloncal antibody is provided in the kit and is validated for Total FVII(a) ELISA assay); rabbit anti-human factor FVII(a) antibody (Stago B.V., antibody is provided in the kit and is validated for Total FVII(a) ELISA assay by the manufacturer)

Influence of HMB-001 on triggering TLT-1 shedding: Primary antibody: polyclonal goat anti-human sTLT-1 (R&D Systems, 842685, LOT# UOK0117021, antibody is provided in the kit and is validated for sTLT-1 ELISA assay by the manufacturer). Secondary antibody: IRDye® 800CW labelled donkey anti-goat IgG secondary antibody (LI-COR, 92632214, LOT# D01007-06).

Assessment of FVII activity and FVIIa plasma levels in GT plasma: Capture: bivalent monoclonal anti-FVIIa specific VHH (UMCU in-house produced, validated for ELISA by Hyseni et al. J Thromb Haemost. 2013 Dec;11(12):2111-7. doi: 10.1111/jth.12427); Detection: primary polyclonal antibody sheep anti-FVII IgG affinity purified (Kordia, SAVFVII-AP, LOT# AP2113-AR2) (validated for ELISA by manufacturer); Secondary antibody: HRP-conjugated rabbit anti-sheep IgG (DAKO, P0163, LOT# 20020223).

FACS: AlexaFluor-647 labelled anti-FVIIa VHH antibodies (UMCU in-house produced), validated for FVIIa binding (Hyseni et al. J Thromb Haemost. 2013 Dec;11(12):2111-7. doi: 10.1111/jth.12427); FITC-labelled mouse anti-P-selectin antibody (clone VI-PL44) (BD Pharmingen, 555523, LOT# 1267266) (validated for FACS); R-PE labelled anti-GPIbα VHH antibodies1 (clone 17, UMCU in-house produced, validated for FACS by Van Dijk et al. J Thromb Haemost. 2023 Apr;21(4):1020-1031. doi: 10.1016/j.jtha.2022.11.039.) AlexaFluor-647 labelled anti-P-selectin VHH antibody1 (clone B10.6, UMCU in-house produced, validated for FACS by Van Dijk et al. J Thromb Haemost. 2023 Apr;21(4):1020-1031. doi: 10.1016/j.jtha.2022.11.039.)) AlexaFluor-488 labelled anti-fibrinogen VHH antibody1 (clone C3, UMCU in-house produced, validated for FACS by Van Dijk et al. J Thromb Haemost. 2023 Apr;21(4):1020-1031. doi: 10.1016/j.jtha.2022.11.039.) AlexaFluor-488- and AlexaFluor-647 labelled isotype control VHH antibody, raised against azo dye RR6, clone R2 (Amino acid sequence derived from crystal structure 1QD0 (Spinelli et al. Biochemistry 15, 39, 1217-22.) produced at UMCU , validated for FACS) AlexaFluor-488 labelled parental TLT-1 antibody (Hemab internal antibody, Lot # ABLOT004). AlexaFluor-488 labelled IgG4 isotype control (Acro Biosystems, DNP-M3, LOT# M3-203AF1-UW)

Effect of HMB-001 on thrombin generation in GT-like platelets: neutralizing mouse anti-TF monoclonal antibody, clone 1F44 (Novo Nordisk A/S, compound# 1212-0000-0127, batch# 7B); neutralizing sheep anti-FVIII polyclonal (Haemtech, PAHFVIII-S, LOT# LL0309-5MG), validated for FVIII inhibition by the manufacturer.

Effect of HMB-001 on FVIIa-dependent fibrin formation on adhered GT and GT-like platelets in flowing blood: AlexaFluor488-conjugated VHH anti-fibrin (clone B12) (UMCU in-house produced), validated in Van Moorsel et al. Thromb Haemost. 2022 May;20(5):1213-1222. doi: 10.1111/jth.15674. MitoTracker™ Orange CMTMRos (Invitrogen, M7510, LOT# 2286848)

Validation

all available information on antibody validation is provided above.

Eukaryotic cell lines

Policy information about cell lines and Sex and Gender in Research

Cell line source(s)	CHO-EBNALT85 and CHO-EBNA cells were from Icosagen; HEK293-Expi cells were from Invitrogen; CHO and ExpiCHO cells were from Gibco.
Authentication	none of the cell lines were authenticated
Mycoplasma contamination	all cell lines were negative for mycoplasma
Commonly misidentified lines (See ICLAC register)	none were used

Animals and other research organisms

Policy information about studies involving animals; ARRIVE guidelines recommended for reporting animal research, and Sex and Gender in Research

Laboratory animals	The mouse model was generated by replacing the mouse treml1 gene with the human variant and subsequently breeding them with F8−/− mice, which were exon 16-disrupted C57Bl/129S mice. Human TLT-1 positive F8−/− mice were identified by polymerase chain reaction. Mice were housed under standard conditions at Novo Nordisk, Måløv, Denmark (20–23 °C, 30%–60% relative humidity, a 12-hour light/dark cycle, and free access to food and water) in environmentally enriched cages. Mice were 12–16 weeks of age at study start. The Cynomolgus monkeys used for PK/PD evaluation were exposed to 12-hour light/dark cycles at Labcorp, United Kingdom (UK). The ambient temperature was maintained at 19–23°C, and the ambient temperature was set to 36–77%. At study start, the age of all animals ranged from 99 – 143 weeks old and they weighed between 2.21 – 4.46 kg
Wild animals	none were used
Reporting on sex	Mice of both sexes were used in approximately equal numbers (50:50). 4 naïve Cynomolgus monkeys (2 females and 2 males) were used for PK/PD analysis.
Field-collected samples	none were used
Ethics oversight	The mice in vivo study was conducted at Novo Nordisk, Denmark. The study was approved by the Danish Animal Experiments Council, the Danish Ministry of Environment and Food, and Novo Nordisk Welfare Body.<br>The non-human primate PK/PD study was conducted at Labcorp, United Kingdom (UK). Labcorp, UK is licensed to undertake animal studies by the UK Home Office. The non-human primate PK/PD study (Study # 8469444) was approved by Labcorp Animal Welfare and Ethics Review Board. Approval from British Animal Council was not required.

Note that full information on the approval of the study protocol must also be provided in the manuscript.

Clinical data

Policy information about clinical studies

All manuscripts should comply with the ICMJE guidelines for publication of clinical research and a completed CONSORT checklist must be included with all submissions.

Clinical trial registration	not applicable
Study protocol	not applicable
Data collection	not applicable
Outcomes	not applicable

Flow Cytometry

Plots

Confirm that:

- [x] The axis labels state the marker and fluorochrome used (e.g. CD4-FITC).
- [x] The axis scales are clearly visible. Include numbers along axes only for bottom left plot of group (a 'group' is an analysis of identical markers).
- [ ] All plots are contour plots with outliers or pseudocolor plots.
- [ ] A numerical value for number of cells or percentage (with statistics) is provided.

Methodology

Sample preparation	All flow cytometry assays were performed in human whole blood, diluted 10-fold in buffer with agonists and antibodies. Samples were fixated and diluted 20-fold before analysis. Samples were subjected to FACS analysis on a BD FACSCanto™ II Flow Cytometer with FACSDiva™ software and platelets were gated based on forward and side scatter, as well as GPIb expression. Results are expressed as median fluorescent intensity (MFI).
Instrument	FACS CantoII
Software	FACSDiva
Cell population abundance	10.000 GPIb-positive events were recorded per sample.
Gating strategy	Platelets were identified in human whole blood based on forward and side scatter, as well as GPIb-positivity. Isotype control antibodies were used to establish cut-off values.

☒ Tick this box to confirm that a figure exemplifying the gating strategy is provided in the Supplementary Information.
